# Two crystal structures of copper(II) complexes and adducts based on the neutral *N*^1^,*N*^1^-dimethyl-*N*^2^-(pyridin-2-ylmeth­yl)ethane-1,2-di­amine ligand

**DOI:** 10.1107/S2056989025006000

**Published:** 2025-07-08

**Authors:** Basam M. Alzoubi, Carola Schulzke

**Affiliations:** a Department of Basic Sciences Al-Huson University College Al-Balqa Applied, University, Irbid Jordan; b Institut für Biochemie, Universität Greifswald, Felix-Hausdorff-Strasse 5, 17489 Greifswald, Germany; National Taras Shevchenko University of Kyiv, Ukraine

**Keywords:** crystal structure, tridentate NNN ligand, copper(II) complexes, complex adduct, inter­molecular inter­actions

## Abstract

From the reaction of copper(II) chloride dihydrate with *N*^1^,*N*^1^-dimethyl-*N*^2^-(pyridin-2-ylmeth­yl)ethane-1,2-di­amine (denoted N^N^N) a new species comprising three distinct complexes crystallized, namely, aqua­chlorido­[*N*^1^,*N*^1^-dimethyl-*N*^2^-(pyridin-2-ylmeth­yl)ethane-1,2-di­amine-κ^3^*N*,*N*′,*N*′′]copper(II)chlorido­[*N*^1^,*N*^1^-dimethyl-*N*^2^-(pyridin-2-ylmeth­yl)ethane-1,2-di­amine-κ^3^*N*,*N*′,*N*′′]copper(II) tetra­chlorido­cuprate(II), [CuCl(C_10_H_17_N_3_)(H_2_O)][CuCl(C_10_H_17_N_3_)][CuCl_4_] or [Cu(N^N^N)(H_2_O)Cl][Cu(N^N^N)Cl_2_][CuCl_4_], the latter two of which form an adduct. This complex was reacted with ammonium thio­cyanate to form [Cu(N^N^N)(NCS)_2_], which crystallized with two independent mol­ecules and a partial water in the asymmetric unit very similarly to the already reported structure of this compound. Both crystal structures show a rather complex set of inter­molecular inter­actions including hydrogen bonding and what may be described as chalcogen bonding, pnictogen bonding and/or coinage bonding. The mol­ecular structures, intra­molecular inter­actions and packing patterns are discussed in detail and the more unusual ones are set into the context of structures available in the literature.

## Abstract 2

Two crystal structures of copper(II) complexes with a tridentate ligand are reported, new [Cu(N^N^N)(H_2_O)Cl][Cu(N^N^N)Cl_2_][CuCl_4_] and already known [Cu(N^N^N)(NCS)_2_]·0.3 H_2_O, (N^N^N = *N^1^*,*N^1^*-dimethyl-*N^2^*-(pyridin-2-ylmeth­yl)ethane-1,2-di­amine), which exhibit a considerable range of inter­molecular inter­actions and notable packing patterns that are being discussed.

## 1. Chemical context

In the first decade of this century, copper has started to become an element of growing inter­est in the context of developing therapeutics against cancer (e.g. Wang & Guo, 2006 ▸; Cvek *et al.*, 2008 ▸; Tardito & Marchiò, 2009 ▸; Marzano *et al.*, 2009 ▸). In a respective review article, a vast number of synthetic attempts and biological studies towards this goal were discussed in detail and some important conclusions were drawn based on this meta-analysis (Santini *et al.*, 2014 ▸). It has emerged, for instance, that the complex should have a good balance between lability and stability and that four- or fivefold coordination geometries were apparently advantageous over sixfold coordination. In view of this, when respective biological investigations were to be addressed, a complex formulation was targeted in one of our labs with a neutral tridentate chelate ligand for some stabilization, while the charge of the copper(II) center was to be neutralized with one or two monodentate ligands that would be more labile and easier to replace in course of the potential inter­action with a biomolecule. Considering that biological copper is quite often coordinated to histidine, *i.e. via* one nitro­gen of the imidazole substituent, an N-donor-rich ligand was chosen. Consequently, *N^1^*,*N^1^*-dimethyl-*N^2^*-(pyridin-2-ylmeth­yl)ethane-1,2-di­amine (denoted N^N^N) was reacted with CuCl_2_ to form the precursor complex with chloride coordination resulting, by chance, in a crystal comprising three differently coordinated copper(II) centers, [Cu(N^N^N)(H_2_O)Cl][Cu(N^N^N)Cl_2_][CuCl_4_] (**1**). Despite the product of the first reaction apparently being a mixture of species, it was further reacted with ammonium thio­cyanate to yield the known targeted complex [Cu(N^N^N)(NCS)_2_] (**2**) (Zhang *et al.*, 2009 ▸).
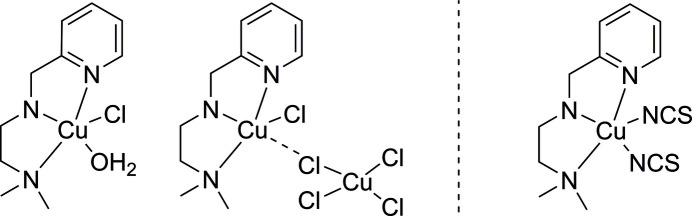


## 2. Structural commentary

**Figure 1:**
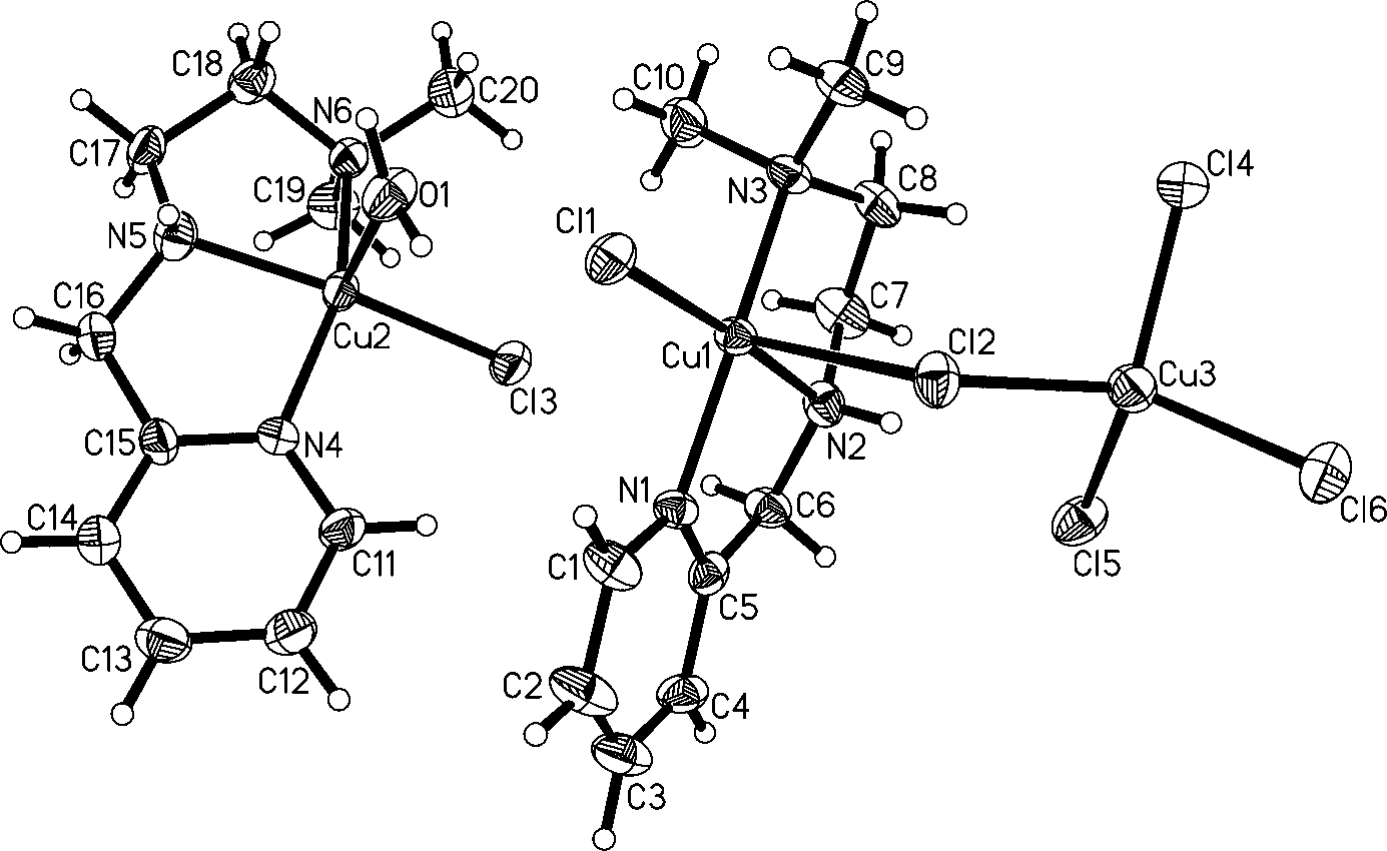
The mol­ecular structure of **1**. Ellipsoids are shown at the 50% probability level.

**Figure 2:**
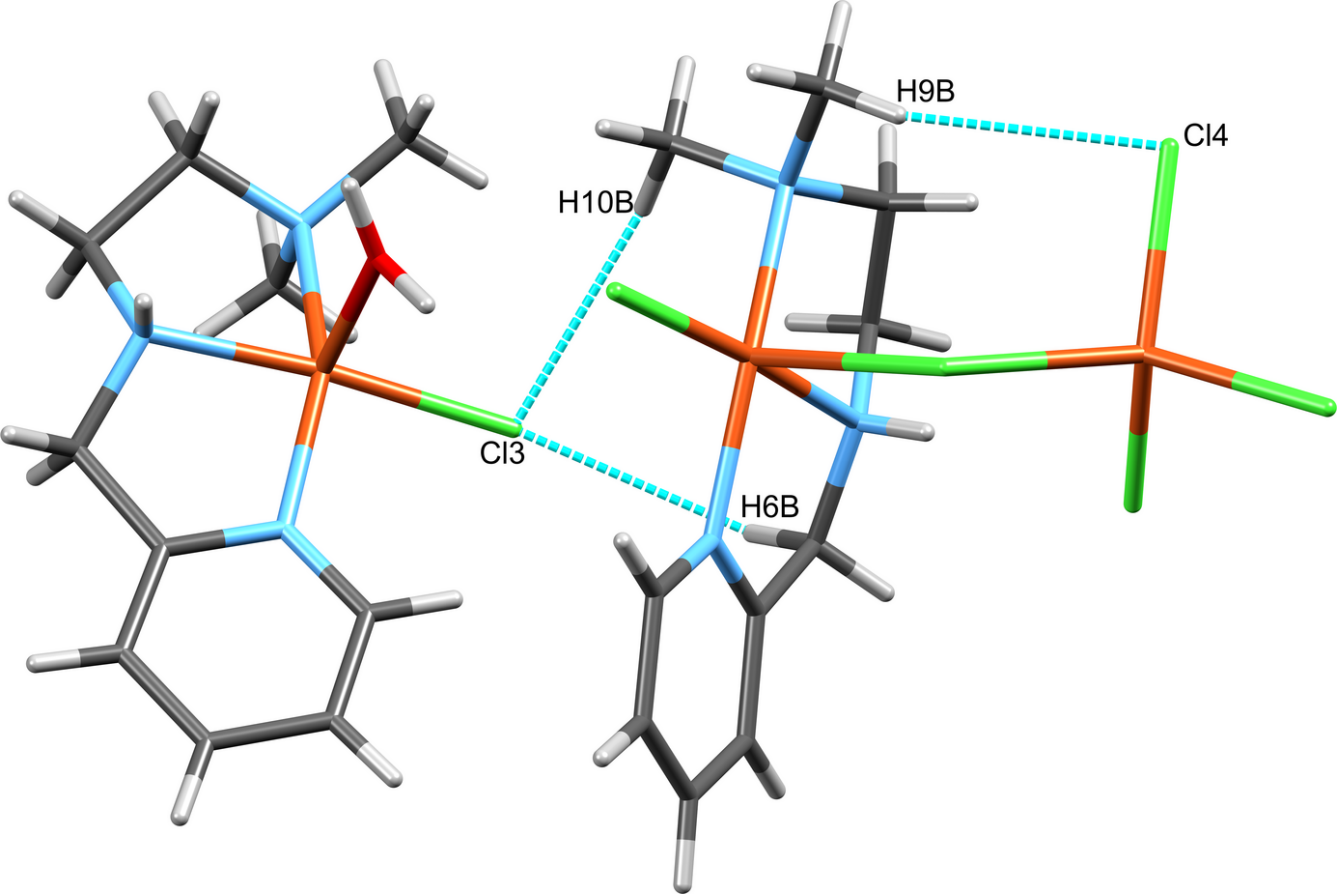
The C—H⋯Cl hydrogen bonding within the asymmetric unit of **1**.

The structure of [Cu(N^N^N)(H_2_O)Cl][Cu(N^N^N)Cl_2_][CuCl_4_] (Fig. 1 ▸) consists of three complexes with copper(II) centers, two of which form an adduct mediated by a chloride ligand (Cl2). [CuCl_4_] has a strongly distorted terahedral coordination geometry around Cu3 with angles ranging from 96.86 (7) to 133.54 (8)° (Table 1 ▸). The geometries around Cu1 and Cu2 are best described as distorted square pyramidal with geometry indices of 0.21 and 0.24, respectively (Addison *et al.*, 1984 ▸) if Cl2 was considered an actual ligand to Cu1. Cu1 is bridged by Cl2 to Cu3. For Cu3 this is a slightly elongated Cu—Cl bond compared to the other three (Table 1 ▸). For Cu1 this bond is rather extended at 2.730 (2) Å, which is substanti­ally longer than the sum of covalent radii (2.34 Å; Cordero *et al.*, 2008 ▸), yet shorter than the sum of the van der Waals radii (3.14 Å; Bondi, 1964 ▸). This contact can therefore be considered a relatively strong coinage (Halldin *et al.*, 2018 ▸) or regium bond (Roy *et al.*, 2024 ▸), terms which are used synonymously and describe the non-covalent inter­action of regions of reduced electron density on the metal center with a nucleophile (*e.g.* a chloride free electron pair). For coinage metals such a hole is typically located perpendicular to the σ-bond skeleton and consequently named a π-hole. All this matches the situation around Cu1 very well considering that the contact lies in the apical position of the square-pyramidal geometry, if this contact is to be considered an actual coordination. Therefore, the assembly of the Cu1 and Cu3 copper centers are best described as adduct rather than as a dinuclear complex. Within the asymmetric unit, three further C—H⋯Cl hydrogen bonds are present, which link all three complexes (Fig. 2 ▸; Table 2 ▸). A Mogul (Bruno *et al.*, 2004 ▸) analysis reveals that there are no unusual metrical parameters in the structure of **1** whatsoever. All values lie well within the observed ranges of related mol­ecular structures deposited in the CSD.

**Figure 3:**
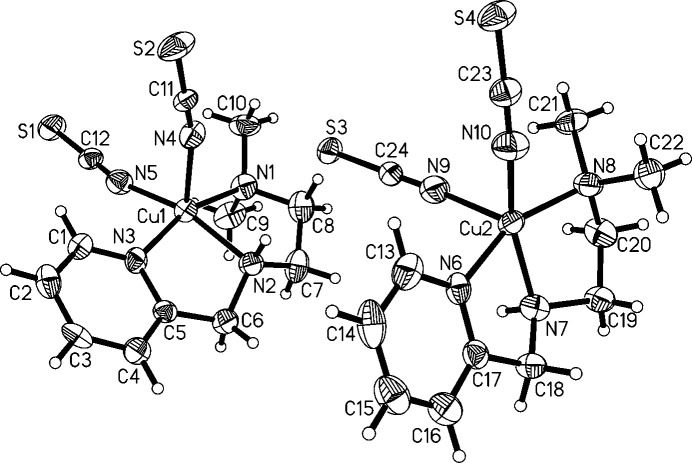
The mol­ecular structure of **2**. The partial water oxygen atom is omitted for clarity. Ellipsoids are shown at the 50% probability level.

The structure of [Cu(N^N^N)(NCS)_2_]·0.3H_2_O (Fig. 3 ▸) is already known, albeit with a slightly different amount of water (refcode VUJGUD; Zhang *et al.*, 2009 ▸). Despite having essentially the same *R*-values as in the published structure, we decided to include our data here for two reasons. Firstly, in the previous study, Zhang and coworkers point out that the two independently refined mol­ecules are two enanti­omers that are co-crystallized, which is based on the distinct conformations of the central amine nitro­gen atoms. A mol­ecular overlay with *Mercury* (Macrae *et al.*, 2020 ▸) allowing inversion results in essentially the same relative locations of all atoms and an r.m.s.d. of 0.4566. In the structure reported here, the two mol­ecules appear slightly less similar to each other. Their overlay results in an r.m.s.d. of 0.7224, which suggests that the mol­ecules have a bit of inherent flexibility. Secondly, and more importantly, the available report is not covering inter­esting features of the packing pattern or inter­mol­ecular inter­actions, as will be discussed in the next section. The compound crystallizes with two independent mol­ecules in the asymmetric unit and bears a site that is occupied by a fraction of water. The occupancy was established by applying SQUEEZE (Spek, 2015 ▸) which resulted in *ca*. 49 electrons per unit cell and a total void size of 200 Å^3^ (or *ca*. six electrons each in eight void sizes of roughly 25 Å^3^). Despite running SQUEEZE, the solvent was not removed from the refinement. Instead, the water oxygen atom was refined with a fixed 0.6 occupancy, which amounts to a 20% increase compared to the known structure. The geometry indices are 0.0 for both complex mol­ecules, supporting square-pyramidal geometries. Despite these very low values there are of course some distortions from ideal sqp geometry. The two mol­ecules of the asymmetric unit are connected by a hydrogen bond, which will be discussed in the following section. With regard to the individual metrical parameters in this mol­ecular structure, the Mogul analysis revealed that none of these were unusual or uncommon. Some features are already discussed in the original publication (Zhang *et al.*, 2009 ▸) and we abstain from a more detailed analysis here.

## 3. Supra­molecular features

Because in both structures there is a large or even very large number of non-classical hydrogen bonds originating from C—H moieties, which face in various directions without clear patterns and some of these are in addition rather long, the following discussion of hydrogen bonds will be focusing on the classical ones and only include some more noteworthy non-classical ones where appropriate (Tables 2 ▸ and 3 ▸). For data on all hydrogen-bonding contacts, please refer to the *Supplementary material*.

**Figure 4:**
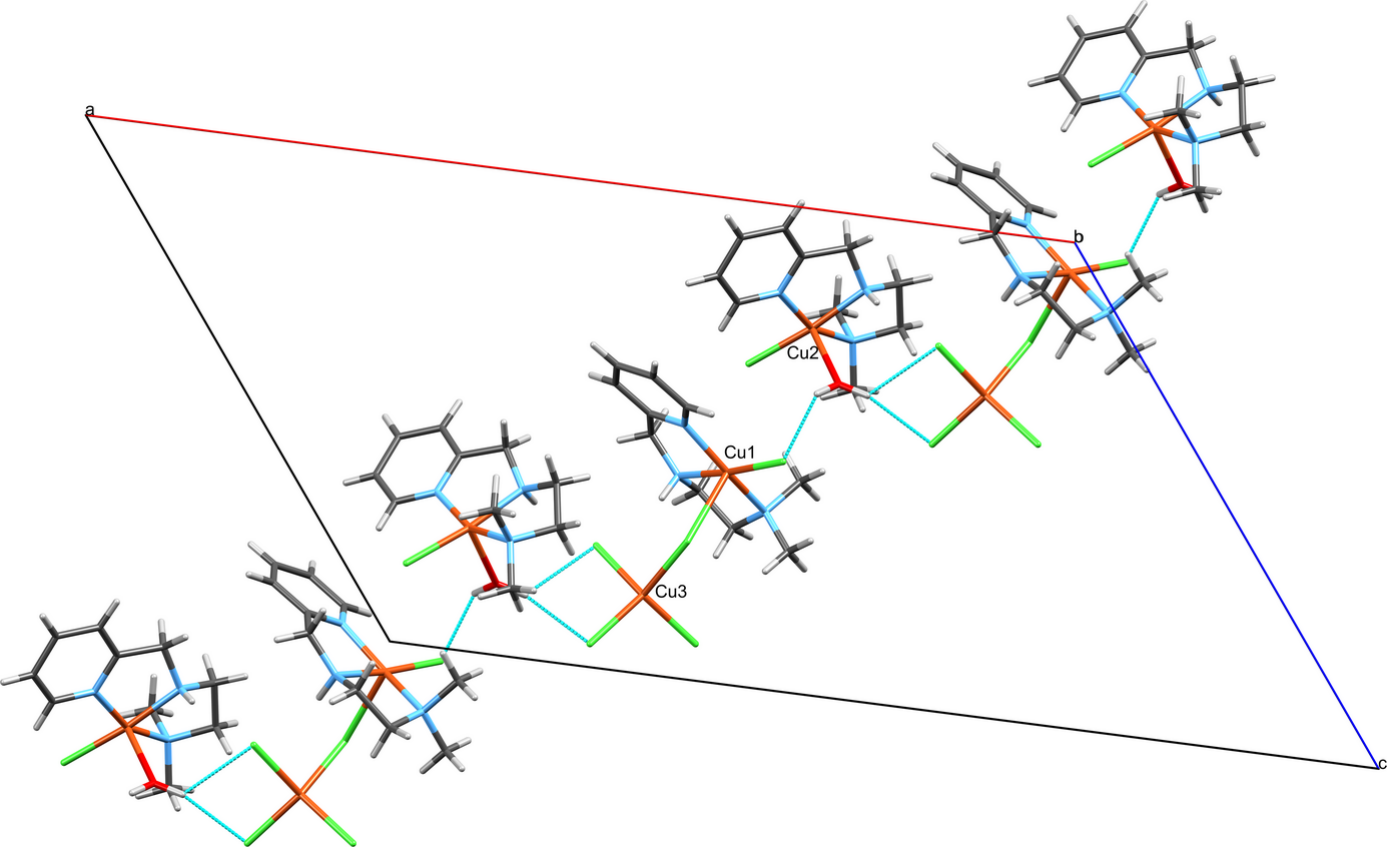
The chain of complex mol­ecules arising from the hydrogen-bonding inter­actions of the water mol­ecule in the crystal of **1**, which protrudes parallel to the *ac*-diagonal viewed along the crystallographic *b*-axis. The copper centers of the asymmetric unit are labeled.

**Figure 5:**
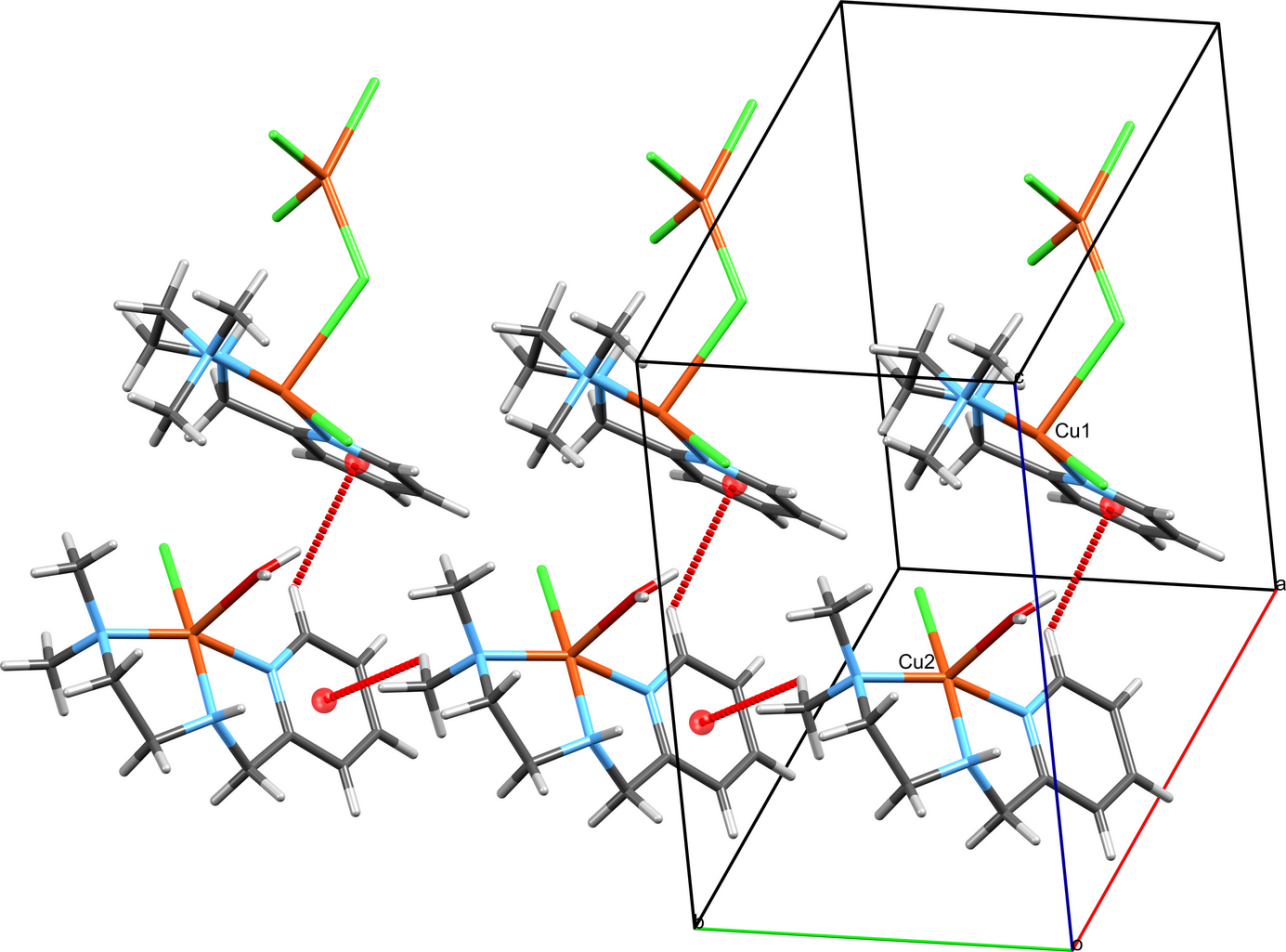
The two C—H⋯π contacts in the structure of **1** of which one connects the Cu1-centered and the Cu2-centered complexes and the other the Cu2-centered complexes with both their adjacent Cu2-centered complexes. The two contacts thereby form unsymmetric ribbons protruding along the crystallographic *b*-axis direction. The respective copper centers of the asymmetric unit are labeled.

**Figure 6:**
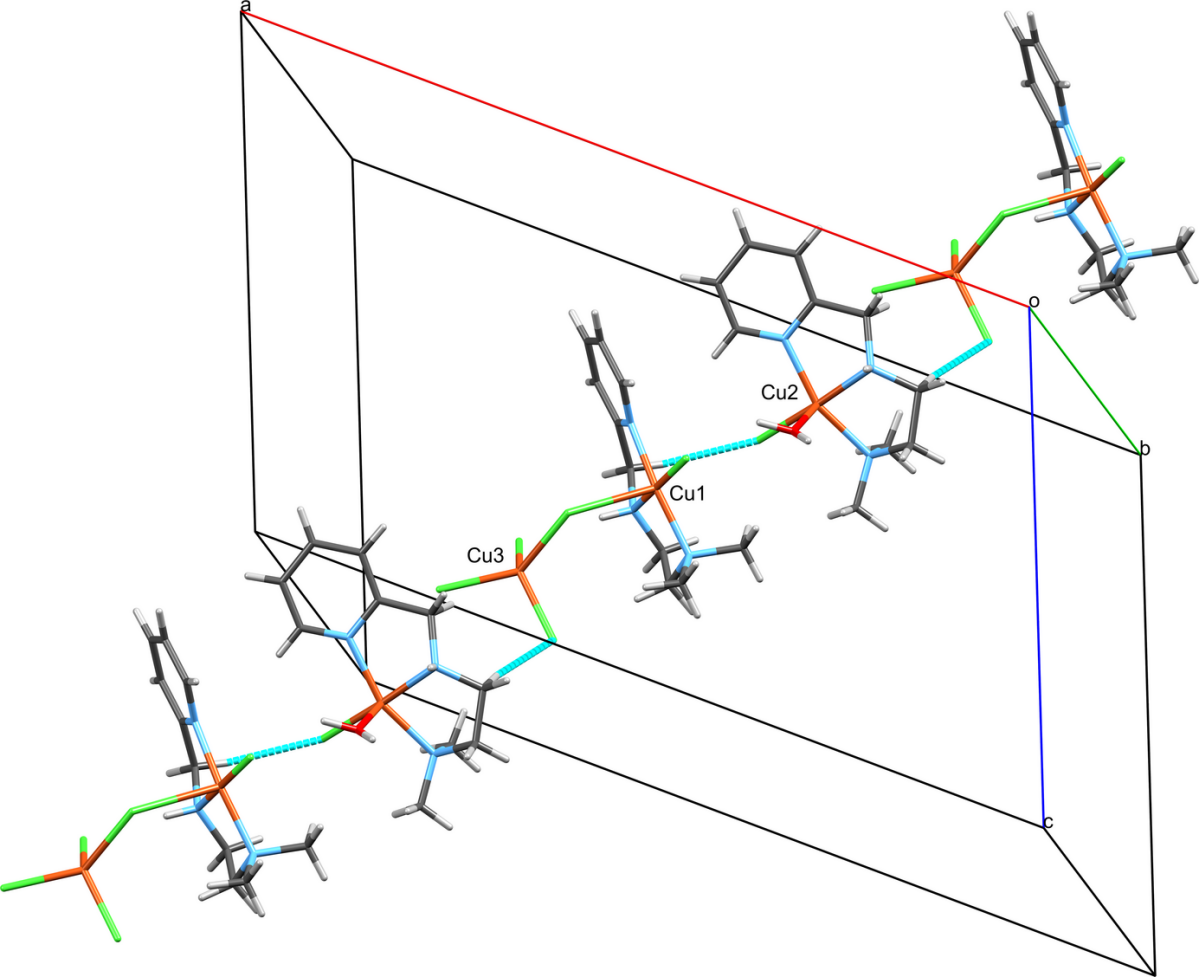
The alternating C—H⋯Cl hydrogen bonds in the structure of **1**, which involve H6*B*/Cl3 and H17*B*/Cl4 and form indefinite chains parallel to the [2
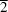
4] unit-cell direction. The copper centers of the asymmetric unit are labeled.

**Figure 7:**
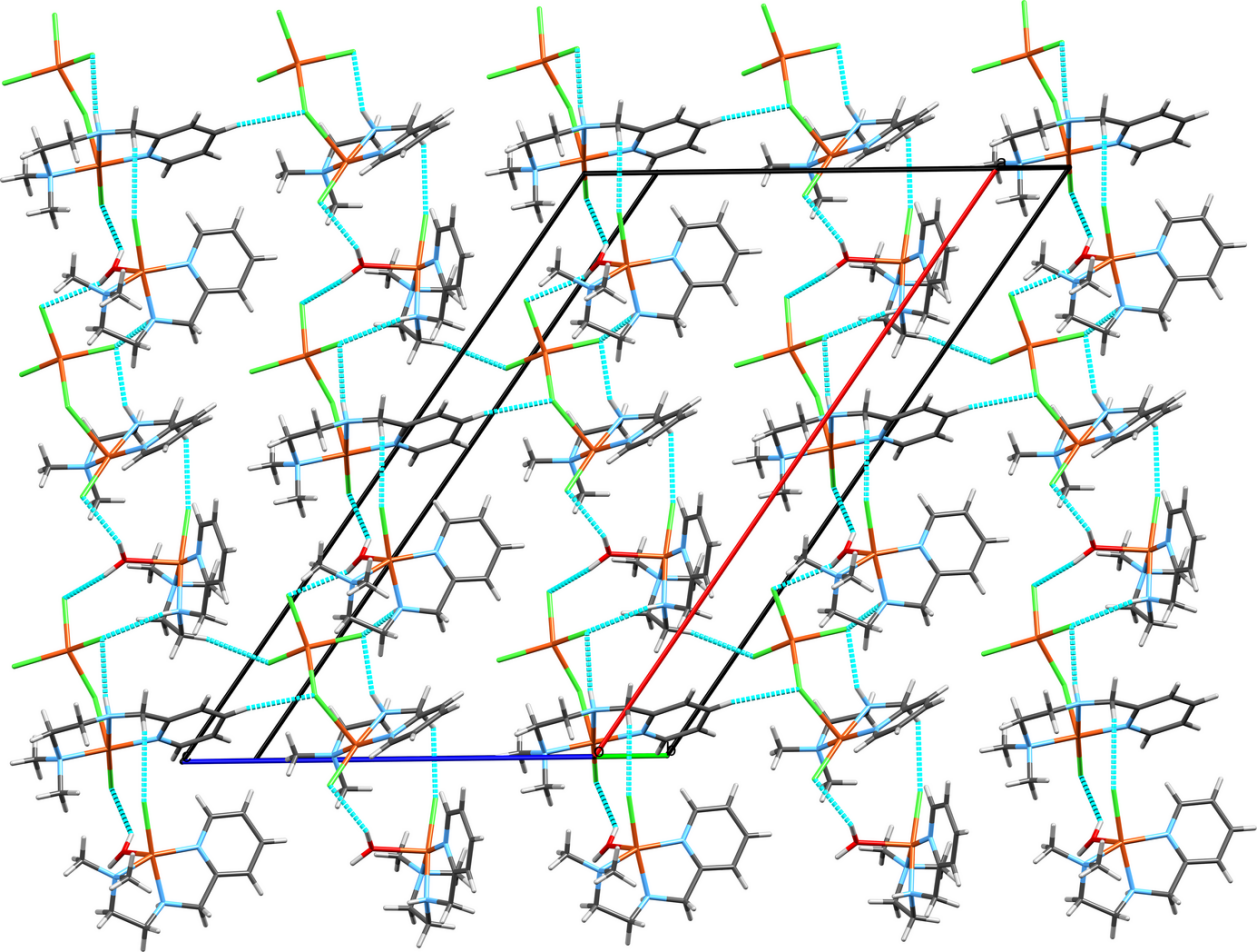
A view of the layer structure consisting of chains formed exclusively by the classical hydrogen bonds (Fig. 4 ▸) and non-classical C—H⋯Cl hydrogen bonds (Fig. 6 ▸) in the crystal of **1** viewed along the Miller vector [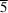
55].

In the crystal of [Cu(N^N^N)(H_2_O)Cl][Cu(N^N^N)Cl_2_][CuCl_4_], hydrogen bonding involving the coordinated water has a particularly important role for the packing. It connects [Cu(N^N^N)(H_2_O)Cl] to both [Cu(N^N^N)Cl_2_] and [CuCl_4_], forming infinite chains that protrude parallel to the *ac* diagonal throughout the crystal (Fig. 4 ▸). One hydrogen (H1*O*) is in contact with two chloride atoms of [CuCl_4_] (Cl5 and Cl6; symmetry code: *x* − 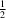, −*y* + 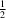, *z* − 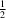) in a bifurcated hydrogen bond and the other (H1*P*) establishes a simple link to [Cu(N^N^N)Cl] (Cl1, within the asymmetric unit). The hydrogen bonds involving N—H merely support the formation of these chains. N2 of [Cu(N^N^N)Cl] forms a bifurcated hydrogen bond to two chlorides, one of which is intra­molecular (Cl2) and the other is inter­molecular (Cl5) to [CuCl_4_] if the two linked centers are considered an adduct. Both hydrogen bonds occur within the asymmetric unit. Additionally, the hydrogen bond originating from N5 of [Cu(N^N^N)(H_2_O)Cl] connects this complex to [CuCl_4_], again involving Cl5, which is thereby the acceptor of three hydrogen bonds. All classical hydrogen bonds are relevant only within one chain but not between chains. There are two further C—H⋯π contacts. One occurs within the asymmetric unit between the pyridine aromatic C11—H11 of the Cu2-centered complex and the pyridine ring of the Cu1 centered complex with a H⋯*Cg* distance of 2.98 Å (*Cg* is the centroid of the pyridine π-ring) and a C—H⋯*Cg* angle of 139°. The second such inter­action links the Cu2-centered complex to the adjacent Cu2-centered complex *via* H19*C* (symmetry operator: *x*, 1 + *y*, *z*) with a H⋯*Cg* distance of 2.91 Å and a C—H⋯*Cg* angle of 118°. In combination they form unsymmetric ribbons protruding along the crystallographic *b*-axis direction (Fig. 5 ▸). Non-classical hydrogen bonds further consolidate the crystal packing (Table 2 ▸). Together with the hydrogen bond C6—H6*B*⋯Cl3 of the asymmetric unit mentioned already in the *Structural commentary* the C17—H17*B*⋯Cl4 contact extending from the asymmetric unit forms infinite chains, which run parallel to the [2
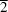
4] unit-cell direction normal (closest Miller vector: [4
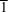
6]; Fig. 6 ▸). In combination with the chains formed by the classical hydrogen bonds, this gives a layer structure with the nearest Miller plane (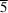
55) (Fig. 7 ▸). Other non-covalent inter­molecular inter­actions between the layers are exclusively C—H-based, non-classical hydrogen bonds. If this type of contact is expanded, it does so in all directions and further clear patterns cannot be distinguished.

**Figure 8:**
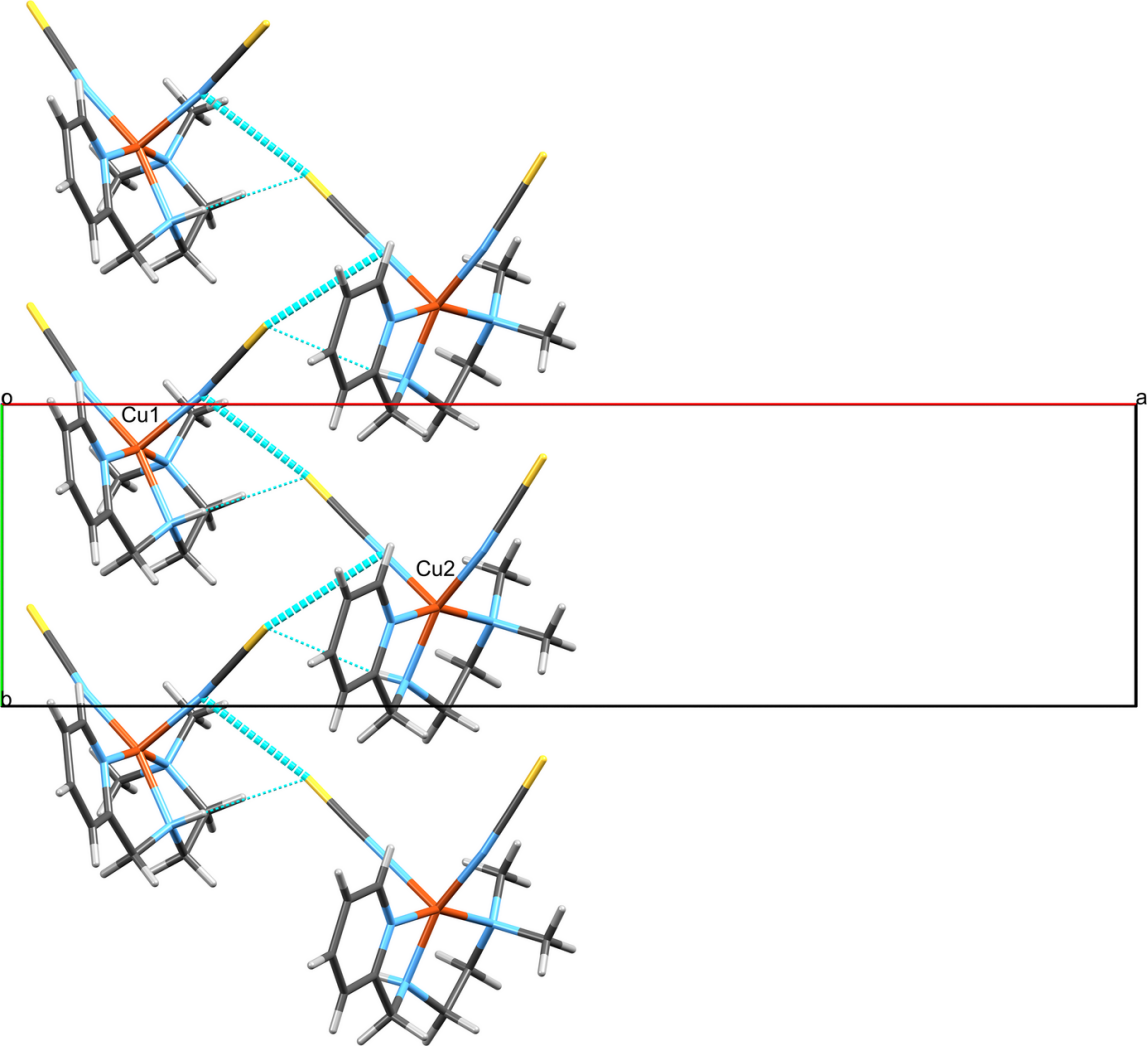
The ribbons in the crystal of **2**, which protrude parallel to the crystallographic *b*-axis viewed along the crystallographic *c*-axis. The copper centers of the asymmetric unit are labelled. The N—H⋯S hydrogen bonds are shown as thin blue dashed lines and the C= S⋯N chalcogen/pnictogen bonds are shown as thick blue dashed lines. Based on the observed respective angles, the non-covalent contact pointing from the Cu2-centered complex to the nitro­gen on the Cu1-centered complex is more likely a chalcogen bond, and the one pointing from the Cu1-centered complex to the nitro­gen of the Cu2-centered complex is more likely a pnictogen bond. The partial water oxygen atom is not shown.

The packing of [Cu(N^N^N)(NCS)_2_]·0.3H_2_O is also largely dominated by inter­molecular hydrogen bonding (Table 3 ▸). The two mol­ecules of the asymmetric unit are linked by the N2—H2*N*⋯S3 hydrogen bond, thus forming a hydrogen-bonded dimer. The dimers are further connected to adjacent dimers by the analogous hydrogen bond N7—H7*N*⋯S2 (symmetry code: *x*, *y* + 1, *z*), which leads to ribbons with a zigzag pattern protruding along the crystallographic *b*-axis direction. The formation of these ribbons appears to be supported by contacts between the terminal thio­cyanate sulfur and a thio­cyanate nitro­gen atom of an adjacent mol­ecule, which at least mirror, but more likely co-define the zigzag motif (Fig. 8 ▸). The respective contact distances are 3.364 (7) Å for S2⋯N9 (symmetry code: *x*, *y* − 1, *z*), and 3.243 (6) Å for S3⋯N4 (within the asymmetric unit). The C=S⋯N angles are notably distinct for these inter­actions at 138.6° for the former and 161.93° for the latter. Such a contact can be considered as a weak chalcogen bond, which is defined as the inter­action of an electropositive region, *i.e.* a hole in the electron density, on a chalcogen atom with a nucleophile and atom-to-atom distances which are longer than the sum of covalent radii and shorter than the sum of van der Waals radii (Aakeroy *et al.*, 2019 ▸; Mahmudov *et al.*, 2022 ▸). An alternative description would be with reversed polarization as a pnictogen bond (Bauzá *et al.*, 2015 ▸; Mahmudov *et al.*, 2020 ▸) in which the nitro­gen is the bond donor bearing a hole (π in this case) and the nucleophilic sulfur atom adopts the role of the bond acceptor. A hole is specified as a σ-hole if it resides on the opposite side of the chalcogen/pnictogen relative to a covalent bond and as a π-hole if it resides in a location which would render the chalcogen/pnictogen bond perpendicular to the covalent bond. For coordinated N-donor thio­cyanate ligands, the formation of chalcogen bonding was described before (refcode VAYGEU; Ghorai *et al.*, 2018 ▸) and the 158.63° C=S⋯*A* angle in the known structure is similar to the one observed here for the two mol­ecules in the asymmetric unit with 161.93°. This value is sufficiently close to the expected 180° for a chalcogen bond with the hole residing on the sulfur atom opposite to the double bond and in between the two lone pairs. The second contact between the sulfur and the nitro­gen of the thio­cyanate ligands extending out of the asymmetric unit has a more acute angle of 138.61°. This would be more in accordance with the ideal 120° angle in the case of an inter­action of a lone pair of sulfur residing in an *sp*^2^-hybridized orbital and a π-hole on the nitro­gen. Therefore it is likely that both types of inter­molecular inter­actions are observed in this structure linking atoms of the same types and chemical environments but with non-covalent bonds of distinct character. The observed ribbons are consequently consolidated by a mixture of inter­molecular inter­actions (hydrogen, chalcogen and pnictogen bonds).

**Figure 9:**
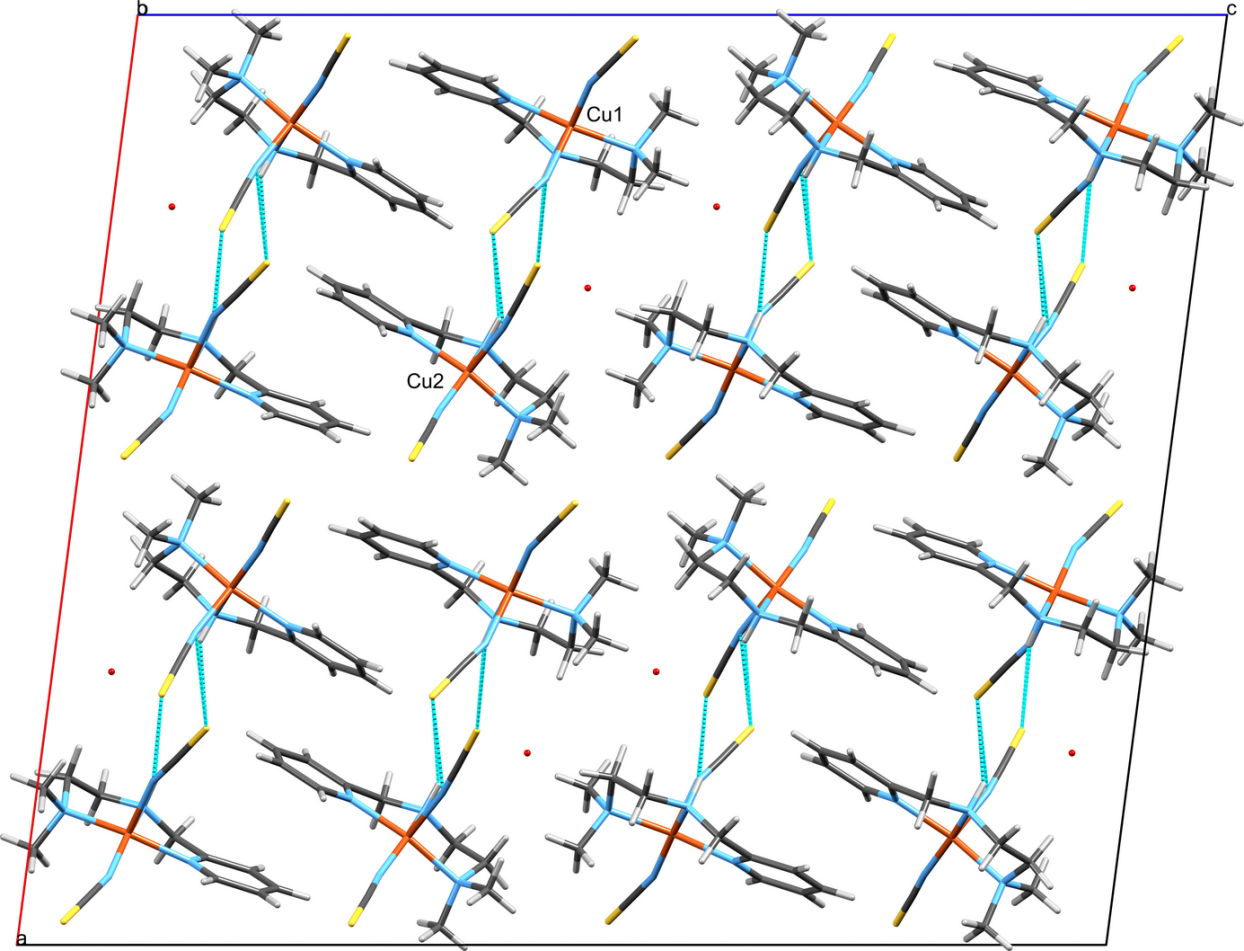
The unit-cell content of **2** including classical N—H⋯S hydrogen bonds and the C=S⋯N chalcogen/pnictogen bonds viewed along the crystallographic *b*-axis. Since *b* is rather short, there is only a single *ac*-layer of mol­ecules in the unit cell. The ribbons, which protrude parallel to the *b*-axis, are represented by two complex mol­ecules each, which are connected by hydrogen bonding. The ribbons are further connected by π–π-stacking to one adjacent ribbon each so that the unit cell holds four pairs of ribbon links. The copper centers of the asymmetric unit are labeled.

Two adjacent ribbons in the *c*-axis direction face each other with the pyridine rings of one ribbon extended toward the other, which is related by inversion symmetry. The resulting off-set π–π-stacking appears as a zipper, which holds the two adjacent ribbons together (Fig. 9 ▸). The centroid–centroid distance for the contact from the Cu1 complex to the Cu2 complex is 3.800 (1) Å, the angle between the planes of the aromatic rings is 9°, and the slippage is 1.654 Å. For the contact from the Cu2 complex to the Cu1 complex these parameters are identical except for the slippage, which is slightly shorter at 1.261 Å. The ribbons are further connected by various C—H⋯*A* contacts, which, apart from the direction of the crystallographic *b*-axis, extend the network in directions *x*, −*y* + 1, *z* + 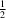 and −*x* + 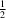, *y* + 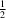, −*z* + 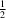.

## 4. Database survey

A search of the CSD database version 2025.1 (Groom *et al.*, 2016 ▸) with ConQuest (Bruno *et al.*, 2002 ▸) for the exact neutral ligand used in the two complexes of this study resulted in 19 hits for complex structures with this ligand employing various metals. Restricting the search to only copper complexes returned nine hits with one mol­ecule appearing thrice, *i.e.* seven different mol­ecules. One of these is the same structure as reported here with essentially identical features (see *Structural commentary* above), apart from the actual occupancy of the co-crystallized water mol­ecule, which, to a certain extent, is a matter of inter­pretation (refcode VUJGUD; Zhang *et al.*, 2009 ▸). The ligand adopts, with the exception of the direction of the pyramidalization of the central amine nitro­gen atom, in principle the same coordination geometry throughout all examples. In all cases the copper centers are in oxidation state (II) and in distorted geometries, which resemble rather a square-pyramidal than a trigonal–bipyramidal arrangement. As complementing ligands there were found a combination of perchlorate and water (BIJQUI; Kumar *et al.*, 2013 ▸), perchlorate and azide (FIFZOK; Sarkar *et al.*, 2005 ▸), two chlorides (FIXLOR, Orton *et al.*, 2023 ▸; MATROP, Raja *et al.*, 2005 ▸; MATROP01, Madhu, 2022 ▸; essentially all the exact same structure despite the different refcodes), two bromides (XEHSOW; Nagarasu *et al.*, 2022 ▸), terephthalic acid forming coordination polymers (WACMAP; Bian *et al.*, 2003 ▸), and finally thio­cyanate as a bridging S,N donor ligand resulting in a dinuclear complex in which each copper center is bound by one N of one SCN^−^ and one S of the other (VUJHAK; Zhang *et al.*, 2009 ▸).

The CSD was further searched for crystal structures of copper complexes with thio­cyanate and any other ligand(s) in which the inter­molecular S⋯N distances lie within the range of 3.0–3.4 Å and may be inter­preted as a weak chalcogen or pnictogen bond, similar to what was observed here for **2**. The distance range was chosen so that it remains shorter than the sum of the van der Waals radii of sulfur and nitro­gen of 3.5 Å (Bondi, 1964 ▸) and substanti­ally longer than the sum of the covalent radii for a single bond of 1.76 Å (Blom & Haaland, 1985 ▸; Cordero *et al.*, 2008 ▸). This returned ten hits (89 if the coordinated metal was unspecified). The observed inter­molecular S⋯N distances range from 3.07 Å (YESZOM; de Geest *et al.*, 2007 ▸) to 3.39 (VUJGUD, Zhang *et al.*, 2009 ▸; QANDIT, Paşaoğlu *et al.*, 2005 ▸). The other structures with inter­molecular distances falling into this range are QANDIT01 (Moncol, 2020 ▸), ABOWEW (Handy *et al.*, 2017 ▸), DEXSAB (Li *et al.*, 2007 ▸), HAGSEQ and HAGSEQ01 (Laussmann *et al.*, 2015 ▸), IZUNOI (Świtlicka *et al.*, 2016 ▸) and OKUMAI (Shen *et al.*, 2003 ▸). The terminal sulfide is in all cases pointing to the side of the nitro­gen of a second thio­cyanate (*i.e.* the respective S⋯N=C moieties are not arranged in line). The C=S⋯N angle, however, appears to have a bit more flexibility which would be in accordance with the observation that apparently such contact can be a chalcogen bond or a pnictogen bond. Presumably both options are generally possible, a terminal σ-hole on sulfur or a π-hole on nitro­gen as the respective bond donor. Considering the relative uniformity of the observed inter­actions, we have reason to speculate that they do indeed play a relevant role for the assembly of the respective crystals.

## 5. Synthesis and crystallization

The ligand *N^1^*,*N^1^*-dimethyl-*N^2^*-(pyridin-2-ylmeth­yl)ethane-1,2-di­amine was obtained from a reaction of *N*,*N*′-di­methyl­ethylenedi­amine (1.32 g, 15 mmol) with pyridine-2-carbaldehyde (1.61 g, 15 mmol) in 30 mL methanol followed by reduction of the inter­mediate Schiff base. Sodium tetra­hydridoborate (0.76 g, 20 mmol) was added to the solution and the mixture was stirred for 3 h under argon. The solution was quenched with 100 ml of 2 *M* NaOH. The mixture was extracted with CH_2_Cl_2_ and the solvent removed *in vacuo* to give the product as a pale-yellow oil. ^1^H NMR (300MHz, CDCl_3_) *δ*_ppm_: 8.55 (*d*, *J* = 4.98 Hz, 1 H), 7.64 (*td*, *J* = 7.66, 1.74 Hz, 1 H), 7.34 (*d*, *J* = 7.76, 1 H), 7.15 (*dd*, *J* = 7.43, 4.95 Hz, 1 H), 3.94 (*s*, 2 H), 2.74 (*t*, *J* = 6.37 Hz, 2 H), 2.67 (*br.s*, 1 H), 2.48 (*t*, *J* = 6.17 Hz, 2 H), 2.46 (*t*, *J* = 6.19 Hz, 11 H), 2.22 (*s*, 6 H).

The blue compound **1** was synthesized by the reaction of the oily ligand product (0.36 g, 2 mmol) and 0.34 g (2 mmol) of copper(II)chloride dihydrate in 10 mL of a mixture of methanol and water (50:50).

The green compound **2** was formed by the reaction of **1** (1.41 g, 2 mmol) with ammonium thio­cyanate (0.3 g, 4 mmol) in 10 mL methanol (non-dried).

Crystals of the complexes were obtained in both cases directly from the mother liquor solutions.

## 6. Refinement

Crystal data, data collection and structure refinement details are summarized in Table 4 ▸. For data solution and refinement the *WinGX* GUI was used (Farrugia, 2012 ▸).

All carbon-bound hydrogen atoms were included using a riding model starting from calculated positions (C_arom_—H 0.95 Å; C_meth­ylene_—H 0.99 Å; C_meth­yl_—H 0.98 Å) with isotropic *U*_iso_(H) values fixed to 1.2 × *U*_eq_ for aromatic and methyl­ene H and to 1.5 × *U*_eq_ for methyl H of the respective parent carbon atom.

In the structure of **1** the N-bound hydrogen atoms were found and refined completely freely. The water hydrogen atoms were located and the O—H and H to H distances were restrained using DFIX with very small allowed deviations so that they could rotate freely but not elongate or shorten their respective distances. The water mol­ecule was therefore refined in a way to find its orientation without force and the respective hydrogen atom locations and their engagement in hydrogen bonding are, hence, defined by the data and factual. The displacement parameters of the water hydrogen atoms were refined freely. Three reflexes were omitted as clear outliers.

In the structure of **2** the N-bound hydrogen atoms were found and constrained with SADI for the N—H distances at a harshened deviation limit of 0.02. With regard to relative orientation and displacement parameters they were refined freely. The water oxygen atom was refined with a fixed occupancy of 0.6, which had been established using SQUEEZE in *PLATON* (Spek, 2015 ▸). With regard to the water hydrogen atoms, they could not be refined in an appropriate strategy based on data. The reason is most plausibly that for the water mol­ecule there are various options for engagement in hydrogen bonding both as donor and as acceptor. For geometric reasons, all these potential hydrogen bonds cannot be served concomitantly. There are, hence, distinct probable orientations of the hydrogen atoms in the crystal lattice and merely fractions of hydrogen occupancies in individual locations of an already not fully occupied water mol­ecule. It was therefore decided to completely abstain from refining the water hydrogen atoms. One reflex was omitted as clear outlier.

## Supplementary Material

Crystal structure: contains datablock(s) 1, 2. DOI: 10.1107/S2056989025006000/nu2011sup1.cif

Structure factors: contains datablock(s) 1. DOI: 10.1107/S2056989025006000/nu20111sup4.hkl

Structure factors: contains datablock(s) 2. DOI: 10.1107/S2056989025006000/nu20112sup5.hkl

CCDC references: 2469615, 2469614

Additional supporting information:  crystallographic information; 3D view; checkCIF report

## Figures and Tables

**Table 1:** Selected geometric parameters (Å, °) for **1**[Chem scheme1]

Cu1—Cl1	2.2331 (18)	Cu3—Cl4	2.235 (2)
Cu1—Cl2	2.730 (2)	Cu3—Cl6	2.238 (2)
Cu2—Cl3	2.258 (2)	Cu3—Cl5	2.269 (2)
Cl2—Cu3	2.251 (2)		
			
Cl4—Cu3—Cl6	100.74 (8)	Cl4—Cu3—Cl5	129.93 (8)
Cl4—Cu3—Cl2	101.80 (8)	Cl6—Cu3—Cl5	98.92 (8)
Cl6—Cu3—Cl2	133.54 (8)	Cl2—Cu3—Cl5	96.86 (7)

**Table 2:** Hydrogen-bond geometry (Å, °) for **1**[Chem scheme1]

*D*—H⋯*A*	*D*—H	H⋯*A*	*D*⋯*A*	*D*—H⋯*A*
C6—H6*B*⋯Cl3	0.99	2.80	3.578 (8)	136
C9—H9*B*⋯Cl4	0.98	2.94	3.827 (8)	152
C10—H10*B*⋯Cl3	0.98	2.85	3.759 (8)	155
N2—H2*N*⋯Cl2	0.91 (9)	2.81 (9)	3.430 (6)	127 (6)
N2—H2*N*⋯Cl5	0.91 (9)	2.39 (8)	3.223 (6)	153 (7)
N5—H5*N*⋯Cl5^i^	0.76 (9)	2.62 (9)	3.352 (7)	161 (8)
C17—H17*B*⋯Cl4^ii^	0.99	2.78	3.755 (8)	168
O1—H1*O*⋯Cl5^i^	0.96 (1)	2.70 (6)	3.358 (6)	127 (5)
O1—H1*O*⋯Cl6^i^	0.96 (1)	2.52 (5)	3.295 (6)	138 (6)
O1—H1*P*⋯Cl1	0.96 (1)	2.37 (7)	3.175 (6)	141 (8)

Symmetry codes: (i) 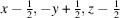; (ii) 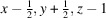.

**Table 3:** Hydrogen-bond geometry (Å, °) for **2**[Chem scheme1]

*D*—H⋯*A*	*D*—H	H⋯*A*	*D*⋯*A*	*D*—H⋯*A*
N2—H2*N*⋯S3	0.91 (7)	2.68 (7)	3.546 (7)	161 (9)
N7—H7*N*⋯S2^i^	0.91 (6)	2.83 (7)	3.623 (7)	146 (6)

Symmetry code: (i) 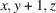.

**Table 4:** Experimental details

	**1**	**2**
Crystal data		
Chemical formula	[CuCl(C_10_H_17_N_3_)(H_2_O)][CuCl(C_10_H_17_N_3_)][CuCl_4_]	C_12_H_17_CuN_5_S_2_·0.3(H_2_O)
*M*_r_	779.87	363.76
Crystal system, space group	Monoclinic, *Cc*	Monoclinic, *C*2/*c*
Temperature (K)	170	170
*a*, *b*, *c* (Å)	29.037 (6), 7.4356 (15), 17.706 (4)	27.626 (6), 7.2787 (15), 32.105 (6)
β (°)	127.34 (3)	97.41 (3)
*V* (Å^3^)	3039.4 (14)	6402 (2)
*Z*	4	16
Radiation type	Mo *K*α	Mo *K*α
μ (mm^−1^)	2.63	1.63
Crystal size (mm)	0.04 × 0.04 × 0.03	0.02 × 0.02 × 0.01

Data collection		
Diffractometer	Stoe IPDS2T	Stoe IPDS2T
Absorption correction	Numerical [face indexed (*X-RED32* and *X-SHAPE*; Stoe & Cie, 2016 ▸)]	Numerical [face indexed (*X-RED32* and *X-SHAPE*; Stoe & Cie, 2016 ▸)]
*T*_min_, *T*_max_	0.993, 0.997	0.843, 0.966
No. of measured, independent and observed [*I* > 2σ(*I*)] reflections	15217, 6832, 5729	22311, 5642, 3563
*R*_int_	0.067	0.094
(sin θ/λ)_max_ (Å^−1^)	0.667	0.595

Refinement		
*R*[*F*^2^ > 2σ(*F*^2^)], *wR*(*F*^2^), *S*	0.039, 0.102, 1.07	0.062, 0.170, 1.04
No. of reflections	6832	5642
No. of parameters	345	378
No. of restraints	5	1
H-atom treatment	H atoms treated by a mixture of independent and constrained refinement	H atoms treated by a mixture of independent and constrained refinement
Δρ_max_, Δρ_min_ (e Å^−3^)	1.07, −1.24	2.09, −0.70
Absolute structure	Flack *x* determined using 2193 quotients [(*I*^+^)−(*I*^−^)]/[(*I*^+^)+(*I*^−^)] (Parsons *et al.*, 2013 ▸)	–
Absolute structure parameter	−0.024 (15)	–

Computer programs: *X-AREA* (Stoe & Cie, 2016 ▸), *SHELXT2018/2* (Sheldrick, 2015*a* ▸), *SHELXL2019/2* (Sheldrick, 2015*b* ▸), *XP* (Sheldrick, 2008 ▸), *CIFTAB* (Sheldrick, 2015*b* ▸) and *publCIF* (Westrip, 2010 ▸).

## supplementary crystallographic information

### Aquachlorido[N1,N1-dimethyl-N2-(pyridin-2-ylmethyl)ethane-1,2-diamine-κ3N,N',N'']copper(II) chlorido[N1,N1-dimethyl-N2-(pyridin-2-ylmethyl)ethane-1,2-diamine-κ3N,N',N'']copper(II) tetrachloridocuprate(II) (1) Crystal data

**Table:**

[CuCl(C_10_H_17_N_3_)(H_2_O)][CuCl(C_10_H_17_N_3_)][CuCl_4_]	*F*(000) = 1580
*M**_r_* = 779.87	*D*_x_ = 1.704 Mg m^−^^3^
Monoclinic, *C**c*	Mo *K*α radiation, λ = 0.71073 Å
*a* = 29.037 (6) Å	Cell parameters from 17421 reflections
*b* = 7.4356 (15) Å	θ = 4.6–59.0°
*c* = 17.706 (4) Å	µ = 2.63 mm^−^^1^
β = 127.34 (3)°	*T* = 170 K
*V* = 3039.4 (14) Å^3^	Block, green
*Z* = 4	0.04 × 0.04 × 0.03 mm

### Aquachlorido[N1,N1-dimethyl-N2-(pyridin-2-ylmethyl)ethane-1,2-diamine-κ3N,N',N'']copper(II) chlorido[N1,N1-dimethyl-N2-(pyridin-2-ylmethyl)ethane-1,2-diamine-κ3N,N',N'']copper(II) tetrachloridocuprate(II) (1) Data collection

**Table:**

Stoe IPDS2T diffractometer	6832 independent reflections
Radiation source: fine-focus sealed tube	5729 reflections with *I* > 2σ(*I*)
Detector resolution: 6.67 pixels mm^-1^	*R*_int_ = 0.067
ω scans	θ_max_ = 28.3°, θ_min_ = 2.3°
Absorption correction: numerical [face indexed (X-Red32 and X-Shape; Stoe & Cie, 2016)]	*h* = −37→38
*T*_min_ = 0.993, *T*_max_ = 0.997	*k* = −9→8
15217 measured reflections	*l* = −23→23

### Aquachlorido[N1,N1-dimethyl-N2-(pyridin-2-ylmethyl)ethane-1,2-diamine-κ3N,N',N'']copper(II) chlorido[N1,N1-dimethyl-N2-(pyridin-2-ylmethyl)ethane-1,2-diamine-κ3N,N',N'']copper(II) tetrachloridocuprate(II) (1) Refinement

**Table:**

Refinement on *F*^2^	Secondary atom site location: difference Fourier map
Least-squares matrix: full	Hydrogen site location: mixed
*R*[*F*^2^ > 2σ(*F*^2^)] = 0.039	H atoms treated by a mixture of independent and constrained refinement
*wR*(*F*^2^) = 0.102	*w* = 1/[σ^2^(*F*_o_^2^) + (0.056*P*)^2^ + 2.5975*P*] where *P* = (*F*_o_^2^ + 2*F*_c_^2^)/3
*S* = 1.07	(Δ/σ)_max_ < 0.001
6832 reflections	Δρ_max_ = 1.07 e Å^−^^3^
345 parameters	Δρ_min_ = −1.24 e Å^−^^3^
5 restraints	Absolute structure: Flack *x* determined using 2193 quotients [(*I*^+^)-(*I*^-^)]/[(*I*^+^)+(*I*^-^)] (Parsons *et al.*, 2013)
Primary atom site location: dual	Absolute structure parameter: −0.024 (15)

### Aquachlorido[N1,N1-dimethyl-N2-(pyridin-2-ylmethyl)ethane-1,2-diamine-κ3N,N',N'']copper(II) chlorido[N1,N1-dimethyl-N2-(pyridin-2-ylmethyl)ethane-1,2-diamine-κ3N,N',N'']copper(II) tetrachloridocuprate(II) (1) Special details

**Table:**

Geometry. All esds (except the esd in the dihedral angle between two l.s. planes) are estimated using the full covariance matrix. The cell esds are taken into account individually in the estimation of esds in distances, angles and torsion angles; correlations between esds in cell parameters are only used when they are defined by crystal symmetry. An approximate (isotropic) treatment of cell esds is used for estimating esds involving l.s. planes.

### Aquachlorido[N1,N1-dimethyl-N2-(pyridin-2-ylmethyl)ethane-1,2-diamine-κ3N,N',N'']copper(II) chlorido[N1,N1-dimethyl-N2-(pyridin-2-ylmethyl)ethane-1,2-diamine-κ3N,N',N'']copper(II) tetrachloridocuprate(II) (1) Fractional atomic coordinates and isotropic or equivalent isotropic displacement parameters (Å^2^)

**Table:**

	*x*	*y*	*z*	*U*_iso_*/*U*_eq_	
Cu1	0.52445 (3)	0.28776 (9)	0.56363 (5)	0.02028 (17)	
Cl1	0.45702 (7)	0.0850 (2)	0.52703 (12)	0.0296 (3)	
N1	0.5411 (2)	0.1896 (7)	0.4765 (4)	0.0211 (11)	
N2	0.5803 (3)	0.4768 (8)	0.5836 (4)	0.0251 (12)	
N3	0.5080 (3)	0.4623 (7)	0.6347 (4)	0.0232 (12)	
C1	0.5275 (4)	0.0235 (9)	0.4389 (5)	0.0300 (15)	
H1	0.505275	−0.052914	0.448528	0.036*	
C2	0.5452 (4)	−0.0387 (11)	0.3866 (6)	0.0395 (18)	
H2	0.535328	−0.156591	0.360543	0.047*	
C3	0.5774 (4)	0.0729 (11)	0.3727 (5)	0.0368 (17)	
H3	0.590427	0.032482	0.337714	0.044*	
C4	0.5903 (3)	0.2430 (10)	0.4101 (5)	0.0299 (15)	
H4	0.611510	0.323231	0.399953	0.036*	
C5	0.5719 (3)	0.2965 (9)	0.4628 (4)	0.0232 (13)	
C6	0.5832 (3)	0.4841 (9)	0.5040 (5)	0.0267 (14)	
H6A	0.621868	0.526304	0.526221	0.032*	
H6B	0.553695	0.568767	0.455071	0.032*	
C7	0.5671 (4)	0.6477 (9)	0.6083 (5)	0.0318 (16)	
H7A	0.533346	0.706466	0.550613	0.038*	
H7B	0.600662	0.730175	0.638709	0.038*	
C8	0.5537 (3)	0.6023 (10)	0.6765 (5)	0.0304 (15)	
H8A	0.589182	0.558163	0.737120	0.036*	
H8B	0.540411	0.711677	0.690050	0.036*	
C9	0.5080 (3)	0.3810 (11)	0.7102 (5)	0.0348 (17)	
H9A	0.501633	0.474623	0.741854	0.052*	
H9B	0.545437	0.322626	0.756567	0.052*	
H9C	0.477005	0.291314	0.682540	0.052*	
C10	0.4502 (3)	0.5435 (11)	0.5627 (5)	0.0359 (17)	
H10A	0.420330	0.450398	0.536903	0.054*	
H10B	0.449548	0.595635	0.511216	0.054*	
H10C	0.442733	0.637878	0.592614	0.054*	
H2N	0.612 (4)	0.418 (12)	0.633 (6)	0.03 (2)*	
Cu2	0.34119 (3)	0.47935 (10)	0.24530 (5)	0.02378 (18)	
Cl2	0.61184 (7)	0.0955 (2)	0.71592 (11)	0.0293 (4)	
N4	0.3545 (2)	0.2858 (8)	0.1810 (4)	0.0249 (12)	
N5	0.2588 (3)	0.3913 (9)	0.1528 (4)	0.0287 (13)	
N6	0.3047 (2)	0.6948 (8)	0.2609 (4)	0.0253 (12)	
H5N	0.254 (4)	0.313 (12)	0.175 (6)	0.03 (2)*	
Cu3	0.69403 (4)	0.22804 (11)	0.83746 (6)	0.0290 (2)	
Cl3	0.43254 (7)	0.5878 (3)	0.33488 (13)	0.0346 (4)	
Cl4	0.66761 (8)	0.3341 (3)	0.92412 (13)	0.0346 (4)	
Cl5	0.71396 (8)	0.3773 (2)	0.74857 (13)	0.0343 (4)	
Cl6	0.78297 (8)	0.1256 (3)	0.95194 (13)	0.0388 (4)	
C11	0.4059 (3)	0.2167 (9)	0.2117 (5)	0.0289 (15)	
H11	0.440020	0.259383	0.269682	0.035*	
C12	0.4106 (3)	0.0876 (10)	0.1623 (6)	0.0323 (15)	
H12	0.447545	0.041181	0.185682	0.039*	
C13	0.3610 (3)	0.0241 (10)	0.0773 (6)	0.0358 (17)	
H13	0.363342	−0.063598	0.040738	0.043*	
C14	0.3083 (3)	0.0919 (11)	0.0476 (5)	0.0328 (16)	
H14	0.273449	0.047755	−0.008845	0.039*	
C15	0.3062 (3)	0.2232 (9)	0.0998 (5)	0.0261 (14)	
C16	0.2508 (3)	0.3049 (10)	0.0711 (5)	0.0311 (15)	
H16A	0.237418	0.395128	0.020574	0.037*	
H16B	0.220748	0.210363	0.045247	0.037*	
C17	0.2179 (3)	0.5335 (9)	0.1314 (5)	0.0301 (15)	
H17A	0.180369	0.481010	0.109968	0.036*	
H17B	0.211055	0.612427	0.080527	0.036*	
C18	0.2443 (3)	0.6393 (10)	0.2217 (5)	0.0312 (15)	
H18A	0.220522	0.747371	0.208336	0.037*	
H18B	0.245204	0.564813	0.268865	0.037*	
C19	0.3032 (4)	0.8410 (10)	0.2020 (6)	0.0363 (17)	
H19A	0.285527	0.948690	0.206491	0.054*	
H19B	0.280287	0.801785	0.135579	0.054*	
H19C	0.342754	0.868728	0.225019	0.054*	
C20	0.3336 (3)	0.7609 (10)	0.3584 (5)	0.0345 (17)	
H20A	0.372373	0.805140	0.383905	0.052*	
H20B	0.336999	0.662635	0.398320	0.052*	
H20C	0.310858	0.858922	0.357729	0.052*	
O1	0.3472 (2)	0.2913 (7)	0.3534 (4)	0.0363 (12)	
H1O	0.323 (2)	0.252 (10)	0.369 (5)	0.05 (3)*	
H1P	0.377 (3)	0.201 (8)	0.379 (7)	0.08 (4)*	

### Aquachlorido[N1,N1-dimethyl-N2-(pyridin-2-ylmethyl)ethane-1,2-diamine-κ3N,N',N'']copper(II) chlorido[N1,N1-dimethyl-N2-(pyridin-2-ylmethyl)ethane-1,2-diamine-κ3N,N',N'']copper(II) tetrachloridocuprate(II) (1) Atomic displacement parameters (Å^2^)

**Table:**

	*U*^11^	*U*^22^	*U*^33^	*U*^12^	*U*^13^	*U*^23^
Cu1	0.0227 (4)	0.0200 (3)	0.0221 (4)	−0.0026 (3)	0.0156 (3)	−0.0020 (3)
Cl1	0.0249 (8)	0.0308 (8)	0.0347 (9)	−0.0063 (6)	0.0190 (7)	−0.0009 (7)
N1	0.026 (3)	0.021 (3)	0.021 (3)	0.004 (2)	0.016 (2)	0.003 (2)
N2	0.026 (3)	0.024 (3)	0.024 (3)	−0.006 (2)	0.015 (3)	−0.003 (2)
N3	0.030 (3)	0.023 (3)	0.021 (3)	0.004 (2)	0.018 (2)	0.000 (2)
C1	0.047 (4)	0.020 (3)	0.029 (3)	0.003 (3)	0.026 (3)	0.001 (3)
C2	0.064 (5)	0.031 (4)	0.033 (4)	0.013 (4)	0.034 (4)	0.006 (3)
C3	0.049 (5)	0.039 (4)	0.030 (4)	0.015 (3)	0.028 (4)	0.006 (3)
C4	0.029 (4)	0.041 (4)	0.029 (3)	0.008 (3)	0.023 (3)	0.009 (3)
C5	0.020 (3)	0.026 (3)	0.021 (3)	0.004 (2)	0.011 (3)	0.006 (2)
C6	0.032 (4)	0.027 (3)	0.029 (3)	−0.006 (3)	0.023 (3)	−0.003 (3)
C7	0.051 (5)	0.015 (3)	0.037 (4)	−0.005 (3)	0.031 (4)	−0.004 (3)
C8	0.040 (4)	0.026 (3)	0.025 (3)	−0.005 (3)	0.020 (3)	−0.006 (3)
C9	0.041 (4)	0.047 (4)	0.025 (3)	0.002 (3)	0.025 (3)	0.003 (3)
C10	0.034 (4)	0.042 (4)	0.027 (4)	0.010 (3)	0.016 (3)	−0.003 (3)
Cu2	0.0174 (3)	0.0258 (4)	0.0234 (4)	0.0003 (3)	0.0100 (3)	−0.0037 (3)
Cl2	0.0268 (8)	0.0300 (9)	0.0259 (8)	−0.0044 (6)	0.0133 (7)	0.0005 (6)
N4	0.024 (3)	0.030 (3)	0.024 (3)	−0.001 (2)	0.015 (2)	−0.004 (2)
N5	0.025 (3)	0.028 (3)	0.029 (3)	0.000 (2)	0.014 (3)	0.000 (2)
N6	0.022 (3)	0.028 (3)	0.024 (3)	0.003 (2)	0.013 (2)	−0.001 (2)
Cu3	0.0284 (4)	0.0323 (4)	0.0284 (4)	−0.0041 (4)	0.0183 (4)	−0.0031 (4)
Cl3	0.0224 (8)	0.0420 (10)	0.0373 (9)	−0.0078 (7)	0.0170 (8)	−0.0135 (7)
Cl4	0.0306 (9)	0.0452 (10)	0.0303 (9)	0.0031 (8)	0.0197 (8)	−0.0026 (8)
Cl5	0.0297 (8)	0.0363 (9)	0.0434 (10)	0.0016 (7)	0.0255 (8)	0.0089 (7)
Cl6	0.0278 (8)	0.0448 (10)	0.0349 (9)	0.0042 (8)	0.0144 (8)	0.0025 (8)
C11	0.023 (3)	0.028 (4)	0.034 (4)	0.003 (3)	0.016 (3)	−0.002 (3)
C12	0.029 (4)	0.030 (4)	0.041 (4)	−0.001 (3)	0.023 (3)	−0.002 (3)
C13	0.042 (4)	0.032 (4)	0.046 (4)	−0.008 (3)	0.033 (4)	−0.011 (3)
C14	0.028 (4)	0.042 (4)	0.028 (4)	−0.006 (3)	0.017 (3)	−0.008 (3)
C15	0.027 (3)	0.028 (3)	0.024 (3)	−0.007 (3)	0.015 (3)	−0.007 (3)
C16	0.024 (3)	0.041 (4)	0.022 (3)	−0.001 (3)	0.011 (3)	−0.006 (3)
C17	0.017 (3)	0.031 (3)	0.031 (4)	0.003 (3)	0.009 (3)	−0.002 (3)
C18	0.026 (3)	0.029 (4)	0.038 (4)	0.004 (3)	0.018 (3)	0.000 (3)
C19	0.041 (4)	0.031 (4)	0.040 (4)	0.005 (3)	0.026 (4)	0.005 (3)
C20	0.033 (4)	0.033 (4)	0.032 (4)	0.007 (3)	0.017 (3)	−0.002 (3)
O1	0.028 (3)	0.042 (3)	0.038 (3)	0.003 (2)	0.019 (2)	0.013 (2)

### Aquachlorido[N1,N1-dimethyl-N2-(pyridin-2-ylmethyl)ethane-1,2-diamine-κ3N,N',N'']copper(II) chlorido[N1,N1-dimethyl-N2-(pyridin-2-ylmethyl)ethane-1,2-diamine-κ3N,N',N'']copper(II) tetrachloridocuprate(II) (1) Geometric parameters (Å, º)

**Table:**

Cu1—N2	2.009 (6)	Cu2—N6	2.030 (6)
Cu1—N1	2.014 (5)	Cu2—Cl3	2.258 (2)
Cu1—N3	2.054 (5)	Cu2—O1	2.290 (5)
Cu1—Cl1	2.2331 (18)	Cl2—Cu3	2.251 (2)
Cu1—Cl2	2.730 (2)	N4—C15	1.343 (9)
N1—C5	1.324 (9)	N4—C11	1.343 (9)
N1—C1	1.343 (9)	N5—C17	1.458 (9)
N2—C6	1.461 (9)	N5—C16	1.467 (9)
N2—C7	1.469 (9)	N6—C20	1.472 (9)
N3—C9	1.468 (9)	N6—C19	1.490 (10)
N3—C8	1.483 (9)	N6—C18	1.499 (9)
N3—C10	1.487 (9)	Cu3—Cl4	2.235 (2)
C1—C2	1.384 (11)	Cu3—Cl6	2.238 (2)
C2—C3	1.378 (12)	Cu3—Cl5	2.269 (2)
C3—C4	1.370 (11)	C11—C12	1.360 (10)
C4—C5	1.384 (10)	C12—C13	1.391 (11)
C5—C6	1.516 (9)	C13—C14	1.377 (11)
C7—C8	1.515 (10)	C14—C15	1.370 (10)
Cu2—N4	2.013 (6)	C15—C16	1.492 (10)
Cu2—N5	2.021 (6)	C17—C18	1.508 (10)
			
N2—Cu1—N1	81.1 (2)	N5—Cu2—Cl3	173.8 (2)
N2—Cu1—N3	85.0 (2)	N6—Cu2—Cl3	96.36 (17)
N1—Cu1—N3	162.0 (2)	N4—Cu2—O1	95.3 (2)
N2—Cu1—Cl1	174.60 (18)	N5—Cu2—O1	86.1 (2)
N1—Cu1—Cl1	96.26 (17)	N6—Cu2—O1	98.6 (2)
N3—Cu1—Cl1	96.65 (17)	Cl3—Cu2—O1	99.76 (15)
N2—Cu1—Cl2	91.43 (18)	Cu3—Cl2—Cu1	121.76 (8)
N1—Cu1—Cl2	93.18 (16)	C15—N4—C11	118.9 (6)
N3—Cu1—Cl2	98.51 (16)	C15—N4—Cu2	114.8 (5)
Cl1—Cu1—Cl2	93.40 (7)	C11—N4—Cu2	126.3 (5)
C5—N1—C1	119.3 (6)	C17—N5—C16	116.5 (6)
C5—N1—Cu1	115.2 (4)	C17—N5—Cu2	110.7 (4)
C1—N1—Cu1	125.3 (5)	C16—N5—Cu2	110.0 (5)
C6—N2—C7	115.7 (6)	C20—N6—C19	109.3 (6)
C6—N2—Cu1	111.6 (4)	C20—N6—C18	108.1 (6)
C7—N2—Cu1	109.2 (4)	C19—N6—C18	110.3 (5)
C9—N3—C8	109.2 (5)	C20—N6—Cu2	116.7 (4)
C9—N3—C10	108.9 (6)	C19—N6—Cu2	106.0 (5)
C8—N3—C10	110.2 (6)	C18—N6—Cu2	106.3 (4)
C9—N3—Cu1	115.2 (5)	Cl4—Cu3—Cl6	100.74 (8)
C8—N3—Cu1	106.3 (4)	Cl4—Cu3—Cl2	101.80 (8)
C10—N3—Cu1	107.0 (4)	Cl6—Cu3—Cl2	133.54 (8)
N1—C1—C2	121.4 (7)	Cl4—Cu3—Cl5	129.93 (8)
C3—C2—C1	119.0 (7)	Cl6—Cu3—Cl5	98.92 (8)
C4—C3—C2	119.1 (7)	Cl2—Cu3—Cl5	96.86 (7)
C3—C4—C5	119.1 (7)	N4—C11—C12	122.0 (7)
N1—C5—C4	122.0 (6)	C11—C12—C13	119.6 (7)
N1—C5—C6	115.9 (6)	C14—C13—C12	118.1 (7)
C4—C5—C6	122.0 (6)	C15—C14—C13	119.7 (7)
N2—C6—C5	108.0 (5)	N4—C15—C14	121.7 (6)
N2—C7—C8	106.6 (6)	N4—C15—C16	115.7 (6)
N3—C8—C7	110.0 (6)	C14—C15—C16	122.7 (6)
N4—Cu2—N5	81.3 (2)	N5—C16—C15	110.5 (5)
N4—Cu2—N6	159.5 (2)	N5—C17—C18	107.0 (5)
N5—Cu2—N6	84.6 (2)	N6—C18—C17	109.6 (6)
N4—Cu2—Cl3	96.09 (17)		
			
C5—N1—C1—C2	0.3 (10)	C15—N4—C11—C12	1.1 (11)
Cu1—N1—C1—C2	−174.1 (5)	Cu2—N4—C11—C12	−178.9 (5)
N1—C1—C2—C3	−0.1 (12)	N4—C11—C12—C13	0.0 (12)
C1—C2—C3—C4	−0.9 (12)	C11—C12—C13—C14	−1.8 (12)
C2—C3—C4—C5	1.6 (11)	C12—C13—C14—C15	2.5 (12)
C1—N1—C5—C4	0.5 (10)	C11—N4—C15—C14	−0.3 (10)
Cu1—N1—C5—C4	175.5 (5)	Cu2—N4—C15—C14	179.7 (6)
C1—N1—C5—C6	177.5 (6)	C11—N4—C15—C16	179.2 (6)
Cu1—N1—C5—C6	−7.5 (7)	Cu2—N4—C15—C16	−0.8 (8)
C3—C4—C5—N1	−1.5 (10)	C13—C14—C15—N4	−1.5 (11)
C3—C4—C5—C6	−178.3 (6)	C13—C14—C15—C16	179.0 (7)
C7—N2—C6—C5	−156.7 (6)	C17—N5—C16—C15	156.4 (6)
Cu1—N2—C6—C5	−31.1 (7)	Cu2—N5—C16—C15	29.4 (7)
N1—C5—C6—N2	25.4 (8)	N4—C15—C16—N5	−19.2 (9)
C4—C5—C6—N2	−157.6 (6)	C14—C15—C16—N5	160.3 (7)
C6—N2—C7—C8	168.2 (6)	C16—N5—C17—C18	−162.7 (6)
Cu1—N2—C7—C8	41.3 (7)	Cu2—N5—C17—C18	−36.1 (7)
C9—N3—C8—C7	162.8 (6)	C20—N6—C18—C17	−167.4 (6)
C10—N3—C8—C7	−77.6 (7)	C19—N6—C18—C17	73.1 (7)
Cu1—N3—C8—C7	38.0 (6)	Cu2—N6—C18—C17	−41.4 (6)
N2—C7—C8—N3	−53.4 (8)	N5—C17—C18—N6	51.7 (8)

### Aquachlorido[N1,N1-dimethyl-N2-(pyridin-2-ylmethyl)ethane-1,2-diamine-κ3N,N',N'']copper(II) chlorido[N1,N1-dimethyl-N2-(pyridin-2-ylmethyl)ethane-1,2-diamine-κ3N,N',N'']copper(II) tetrachloridocuprate(II) (1) Hydrogen-bond geometry (Å, º)

**Table:**

*D*—H···*A*	*D*—H	H···*A*	*D*···*A*	*D*—H···*A*
C1—H1···Cl1	0.95	2.70	3.272 (8)	119
C4—H4···Cl4^i^	0.95	2.91	3.783 (8)	153
C6—H6*B*···Cl3	0.99	2.80	3.578 (8)	136
C7—H7*B*···Cl2^ii^	0.99	2.97	3.661 (7)	128
C9—H9*B*···Cl2	0.98	2.96	3.637 (8)	127
C9—H9*B*···Cl4	0.98	2.94	3.827 (8)	152
C9—H9*C*···Cl1	0.98	2.89	3.424 (8)	116
C10—H10*A*···Cl1	0.98	2.96	3.494 (8)	115
C10—H10*B*···Cl3	0.98	2.85	3.759 (8)	155
N2—H2*N*···Cl2	0.91 (9)	2.81 (9)	3.430 (6)	127 (6)
N2—H2*N*···Cl5	0.91 (9)	2.39 (8)	3.223 (6)	153 (7)
N5—H5*N*···Cl5^iii^	0.76 (9)	2.62 (9)	3.352 (7)	161 (8)
C11—H11···Cl3	0.95	2.77	3.305 (7)	117
C16—H16*A*···Cl6^iv^	0.99	2.85	3.661 (8)	140
C17—H17*B*···Cl4^iv^	0.99	2.78	3.755 (8)	168
C18—H18*A*···Cl5^v^	0.99	2.91	3.799 (7)	149
C19—H19*A*···Cl5^v^	0.98	2.90	3.791 (8)	151
C19—H19*C*···Cl3	0.98	2.97	3.531 (8)	118
C20—H20*A*···Cl3	0.98	2.87	3.395 (8)	114
C20—H20*C*···Cl5^v^	0.98	2.98	3.860 (8)	150
O1—H1*O*···Cl5^iii^	0.96 (1)	2.70 (6)	3.358 (6)	127 (5)
O1—H1*O*···Cl6^iii^	0.96 (1)	2.52 (5)	3.295 (6)	138 (6)
O1—H1*P*···Cl1	0.96 (1)	2.37 (7)	3.175 (6)	141 (8)
C1—H1···Cl1	0.95	2.70	3.272 (8)	119
C4—H4···Cl4^i^	0.95	2.91	3.783 (8)	153
C6—H6*B*···Cl3	0.99	2.80	3.578 (8)	136
C7—H7*B*···Cl2^ii^	0.99	2.97	3.661 (7)	128
C9—H9*B*···Cl2	0.98	2.96	3.637 (8)	127
C9—H9*B*···Cl4	0.98	2.94	3.827 (8)	152
C9—H9*C*···Cl1	0.98	2.89	3.424 (8)	116
C10—H10*A*···Cl1	0.98	2.96	3.494 (8)	115
C10—H10*B*···Cl3	0.98	2.85	3.759 (8)	155
N2—H2*N*···Cl2	0.91 (9)	2.81 (9)	3.430 (6)	127 (6)
N2—H2*N*···Cl5	0.91 (9)	2.39 (8)	3.223 (6)	153 (7)
N5—H5*N*···Cl5^iii^	0.76 (9)	2.62 (9)	3.352 (7)	161 (8)
C11—H11···Cl3	0.95	2.77	3.305 (7)	117
C16—H16*A*···Cl6^iv^	0.99	2.85	3.661 (8)	140
C17—H17*B*···Cl4^iv^	0.99	2.78	3.755 (8)	168
C18—H18*A*···Cl5^v^	0.99	2.91	3.799 (7)	149
C19—H19*A*···Cl5^v^	0.98	2.90	3.791 (8)	151
C19—H19*C*···Cl3	0.98	2.97	3.531 (8)	118
C20—H20*A*···Cl3	0.98	2.87	3.395 (8)	114
C20—H20*C*···Cl5^v^	0.98	2.98	3.860 (8)	150
O1—H1*O*···Cl5^iii^	0.96 (1)	2.70 (6)	3.358 (6)	127 (5)
O1—H1*O*···Cl6^iii^	0.96 (1)	2.52 (5)	3.295 (6)	138 (6)
O1—H1*P*···Cl1	0.96 (1)	2.37 (7)	3.175 (6)	141 (8)

Symmetry codes: (i) *x*, −*y*+1, *z*−1/2; (ii) *x*, *y*+1, *z*; (iii) *x*−1/2, −*y*+1/2, *z*−1/2; (iv) *x*−1/2, *y*+1/2, *z*−1; (v) *x*−1/2, −*y*+3/2, *z*−1/2.

### (2) Crystal data

**Table:**

C_12_H_17_CuN_5_S_2_·0.3(H_2_O)	*F*(000) = 2998
*M**_r_* = 363.76	*D*_x_ = 1.510 Mg m^−^^3^
Monoclinic, *C*2/*c*	Mo *K*α radiation, λ = 0.71073 Å
*a* = 27.626 (6) Å	Cell parameters from 34080 reflections
*b* = 7.2787 (15) Å	θ = 3.0–59.2°
*c* = 32.105 (6) Å	µ = 1.63 mm^−^^1^
β = 97.41 (3)°	*T* = 170 K
*V* = 6402 (2) Å^3^	Needle, green
*Z* = 16	0.02 × 0.02 × 0.01 mm

### (2) Data collection

**Table:**

Stoe IPDS2T diffractometer	5642 independent reflections
Radiation source: fine-focus sealed tube	3563 reflections with *I* > 2σ(*I*)
Detector resolution: 6.67 pixels mm^-1^	*R*_int_ = 0.094
ω scans	θ_max_ = 25.0°, θ_min_ = 1.5°
Absorption correction: numerical [face indexed (X-Red32 and X-Shape; Stoe & Cie, 2016)]	*h* = −32→32
*T*_min_ = 0.843, *T*_max_ = 0.966	*k* = −8→8
22311 measured reflections	*l* = −37→38

### (2) Refinement

**Table:**

Refinement on *F*^2^	Primary atom site location: dual
Least-squares matrix: full	Secondary atom site location: difference Fourier map
*R*[*F*^2^ > 2σ(*F*^2^)] = 0.062	Hydrogen site location: mixed
*wR*(*F*^2^) = 0.170	H atoms treated by a mixture of independent and constrained refinement
*S* = 1.04	*w* = 1/[σ^2^(*F*_o_^2^) + (0.0701*P*)^2^ + 34.5724*P*] where *P* = (*F*_o_^2^ + 2*F*_c_^2^)/3
5642 reflections	(Δ/σ)_max_ = 0.001
378 parameters	Δρ_max_ = 2.09 e Å^−^^3^
1 restraint	Δρ_min_ = −0.70 e Å^−^^3^

### (2) Special details

**Table:**

Geometry. All esds (except the esd in the dihedral angle between two l.s. planes) are estimated using the full covariance matrix. The cell esds are taken into account individually in the estimation of esds in distances, angles and torsion angles; correlations between esds in cell parameters are only used when they are defined by crystal symmetry. An approximate (isotropic) treatment of cell esds is used for estimating esds involving l.s. planes.

### (2) Fractional atomic coordinates and isotropic or equivalent isotropic displacement parameters (Å^2^)

**Table:**

	*x*	*y*	*z*	*U*_iso_*/*U*_eq_	Occ. (<1)
Cu1	0.11991 (3)	0.14704 (11)	0.41101 (2)	0.0284 (2)	
S1	0.02525 (7)	−0.3246 (3)	0.45978 (7)	0.0471 (5)	
S2	0.23285 (8)	−0.2601 (3)	0.35186 (8)	0.0529 (6)	
N1	0.1449 (2)	0.1939 (9)	0.47301 (17)	0.0357 (14)	
N2	0.1488 (2)	0.3961 (8)	0.40076 (18)	0.0337 (14)	
N3	0.09000 (19)	0.1843 (8)	0.35123 (17)	0.0297 (13)	
N4	0.1760 (2)	−0.0311 (9)	0.39436 (19)	0.0374 (14)	
N5	0.0738 (2)	−0.0454 (9)	0.42358 (19)	0.0369 (14)	
C1	0.0712 (2)	0.0549 (10)	0.3250 (2)	0.0312 (15)	
H1	0.067888	−0.065631	0.335657	0.037*	
C2	0.0564 (3)	0.0865 (12)	0.2836 (2)	0.0391 (18)	
H2	0.043179	−0.009991	0.265665	0.047*	
C3	0.0609 (2)	0.2615 (11)	0.2681 (2)	0.0346 (17)	
H3	0.051070	0.286482	0.239185	0.041*	
C4	0.0798 (2)	0.4000 (10)	0.2946 (2)	0.0337 (16)	
H4	0.082858	0.521269	0.284321	0.040*	
C5	0.0944 (2)	0.3587 (9)	0.3368 (2)	0.0275 (14)	
C6	0.1163 (3)	0.4923 (10)	0.3683 (2)	0.0368 (17)	
H6A	0.134888	0.585811	0.354577	0.044*	
H6B	0.090204	0.555810	0.381196	0.044*	
C7	0.1609 (3)	0.4914 (11)	0.4414 (2)	0.0421 (19)	
H7A	0.131469	0.550880	0.449799	0.051*	
H7B	0.185910	0.587142	0.439095	0.051*	
C8	0.1801 (3)	0.3497 (12)	0.4731 (2)	0.0429 (19)	
H8A	0.211865	0.303048	0.466406	0.051*	
H8B	0.185367	0.406156	0.501349	0.051*	
C9	0.1036 (3)	0.2481 (12)	0.4960 (2)	0.046 (2)	
H9A	0.087081	0.355691	0.482396	0.068*	
H9B	0.080382	0.146058	0.495553	0.068*	
H9C	0.116137	0.278220	0.525137	0.068*	
C10	0.1689 (3)	0.0320 (11)	0.4941 (2)	0.047 (2)	
H10A	0.196246	−0.005872	0.479454	0.071*	
H10B	0.181128	0.063293	0.523299	0.071*	
H10C	0.145373	−0.068869	0.493715	0.071*	
C11	0.1992 (2)	−0.1237 (9)	0.3761 (2)	0.0309 (15)	
C12	0.0539 (2)	−0.1618 (10)	0.4384 (2)	0.0304 (15)	
H2N	0.177 (3)	0.356 (16)	0.393 (3)	0.10 (4)*	
Cu2	0.38379 (3)	0.67227 (11)	0.34576 (3)	0.0292 (2)	
S3	0.26967 (6)	0.2390 (2)	0.39685 (6)	0.0332 (4)	
S4	0.47701 (7)	0.1765 (3)	0.30465 (7)	0.0436 (5)	
N6	0.34344 (19)	0.7200 (8)	0.28949 (17)	0.0299 (13)	
N7	0.3559 (2)	0.9188 (9)	0.35779 (18)	0.0349 (14)	
N8	0.4325 (2)	0.7206 (8)	0.39821 (19)	0.0351 (14)	
N9	0.3360 (2)	0.4933 (9)	0.3731 (2)	0.0481 (17)	
N10	0.4249 (2)	0.4835 (9)	0.3244 (2)	0.0452 (16)	
C13	0.3321 (3)	0.5949 (12)	0.2593 (2)	0.0439 (19)	
H13	0.342670	0.471496	0.263993	0.053*	
C14	0.3051 (3)	0.6423 (14)	0.2210 (2)	0.050 (2)	
H14	0.296818	0.552058	0.199881	0.059*	
C15	0.2906 (3)	0.8223 (14)	0.2144 (2)	0.049 (2)	
H15	0.272909	0.858424	0.188322	0.058*	
C16	0.3020 (3)	0.9483 (12)	0.2456 (2)	0.0398 (18)	
H16	0.292003	1.072660	0.241704	0.048*	
C17	0.3283 (2)	0.8923 (10)	0.2830 (2)	0.0308 (15)	
C18	0.3436 (3)	1.0233 (10)	0.3187 (2)	0.0329 (16)	
H18A	0.372248	1.095469	0.312628	0.039*	
H18B	0.316627	1.109908	0.321737	0.039*	
C19	0.3911 (3)	1.0146 (10)	0.3899 (2)	0.0373 (18)	
H19A	0.417572	1.072627	0.376493	0.045*	
H19B	0.374222	1.111235	0.404293	0.045*	
C20	0.4115 (3)	0.8719 (11)	0.4206 (2)	0.0415 (19)	
H20A	0.437067	0.926835	0.441307	0.050*	
H20B	0.385317	0.823791	0.435957	0.050*	
C21	0.4405 (3)	0.5567 (11)	0.4260 (3)	0.047 (2)	
H21A	0.454422	0.456984	0.410875	0.070*	
H21B	0.463003	0.588413	0.451112	0.070*	
H21C	0.409224	0.516901	0.434367	0.070*	
C22	0.4802 (3)	0.7751 (11)	0.3860 (3)	0.0444 (19)	
H22A	0.493666	0.673191	0.371156	0.067*	
H22B	0.475952	0.882569	0.367491	0.067*	
H22C	0.502694	0.805609	0.411198	0.067*	
C23	0.4471 (3)	0.3553 (10)	0.3167 (2)	0.0334 (16)	
C24	0.3084 (2)	0.3906 (10)	0.3834 (2)	0.0299 (15)	
H7N	0.328 (3)	0.890 (11)	0.368 (2)	0.05 (2)*	
O1	0.2063 (4)	0.3173 (18)	0.0543 (4)	0.087 (4)	0.6

### (2) Atomic displacement parameters (Å^2^)

**Table:**

	*U*^11^	*U*^22^	*U*^33^	*U*^12^	*U*^13^	*U*^23^
Cu1	0.0302 (5)	0.0282 (5)	0.0272 (4)	−0.0011 (4)	0.0052 (3)	−0.0005 (4)
S1	0.0456 (11)	0.0339 (11)	0.0619 (13)	−0.0078 (9)	0.0072 (9)	0.0164 (10)
S2	0.0525 (13)	0.0291 (11)	0.0831 (16)	0.0058 (9)	0.0316 (12)	−0.0050 (10)
N1	0.046 (3)	0.037 (4)	0.025 (3)	0.002 (3)	0.007 (3)	−0.005 (3)
N2	0.042 (4)	0.029 (3)	0.027 (3)	0.003 (3)	−0.005 (3)	−0.003 (3)
N3	0.024 (3)	0.032 (3)	0.034 (3)	−0.007 (2)	0.005 (2)	0.004 (3)
N4	0.039 (4)	0.032 (3)	0.042 (4)	−0.005 (3)	0.009 (3)	−0.003 (3)
N5	0.036 (3)	0.038 (4)	0.037 (3)	0.000 (3)	0.006 (3)	0.008 (3)
C1	0.034 (4)	0.028 (4)	0.032 (4)	−0.003 (3)	0.007 (3)	−0.002 (3)
C2	0.030 (4)	0.052 (5)	0.036 (4)	−0.005 (3)	0.006 (3)	−0.011 (4)
C3	0.024 (4)	0.050 (5)	0.028 (4)	0.010 (3)	0.000 (3)	0.010 (3)
C4	0.032 (4)	0.035 (4)	0.034 (4)	−0.003 (3)	0.007 (3)	0.005 (3)
C5	0.023 (3)	0.028 (4)	0.032 (4)	0.001 (3)	0.005 (3)	0.004 (3)
C6	0.034 (4)	0.031 (4)	0.045 (4)	0.000 (3)	0.004 (3)	0.000 (3)
C7	0.046 (5)	0.046 (5)	0.036 (4)	−0.012 (4)	0.011 (3)	−0.015 (4)
C8	0.048 (5)	0.052 (5)	0.028 (4)	−0.006 (4)	0.004 (3)	−0.011 (4)
C9	0.064 (5)	0.042 (5)	0.034 (4)	0.006 (4)	0.021 (4)	−0.001 (4)
C10	0.065 (5)	0.038 (5)	0.035 (4)	0.017 (4)	−0.009 (4)	0.002 (4)
C11	0.032 (4)	0.019 (4)	0.041 (4)	0.000 (3)	0.004 (3)	0.001 (3)
C12	0.038 (4)	0.026 (4)	0.027 (3)	0.003 (3)	0.004 (3)	0.004 (3)
Cu2	0.0316 (5)	0.0220 (4)	0.0343 (5)	0.0005 (4)	0.0049 (3)	0.0013 (4)
S3	0.0310 (9)	0.0282 (9)	0.0415 (10)	−0.0054 (7)	0.0086 (8)	0.0056 (8)
S4	0.0495 (11)	0.0288 (10)	0.0568 (12)	0.0055 (9)	0.0227 (9)	−0.0018 (9)
N6	0.027 (3)	0.035 (4)	0.027 (3)	−0.003 (2)	0.005 (2)	−0.010 (3)
N7	0.034 (3)	0.036 (4)	0.034 (3)	0.005 (3)	0.005 (3)	0.003 (3)
N8	0.032 (3)	0.031 (3)	0.042 (3)	0.006 (3)	0.005 (3)	0.005 (3)
N9	0.032 (4)	0.038 (4)	0.074 (5)	0.001 (3)	0.007 (3)	0.012 (4)
N10	0.047 (4)	0.039 (4)	0.051 (4)	0.010 (3)	0.011 (3)	0.002 (3)
C13	0.048 (5)	0.038 (5)	0.047 (5)	−0.007 (4)	0.009 (4)	−0.009 (4)
C14	0.047 (5)	0.072 (7)	0.030 (4)	−0.020 (4)	0.004 (3)	−0.020 (4)
C15	0.043 (4)	0.069 (6)	0.033 (4)	−0.002 (4)	0.001 (3)	0.005 (4)
C16	0.044 (4)	0.047 (5)	0.028 (4)	0.001 (4)	0.004 (3)	0.003 (3)
C17	0.024 (3)	0.033 (4)	0.035 (4)	−0.001 (3)	0.005 (3)	−0.001 (3)
C18	0.038 (4)	0.030 (4)	0.030 (4)	0.005 (3)	0.002 (3)	0.002 (3)
C19	0.045 (4)	0.029 (4)	0.034 (4)	0.009 (3)	−0.008 (3)	−0.014 (3)
C20	0.043 (4)	0.047 (5)	0.033 (4)	0.010 (4)	0.002 (3)	−0.007 (4)
C21	0.055 (5)	0.032 (4)	0.051 (5)	0.001 (4)	−0.003 (4)	0.019 (4)
C22	0.036 (4)	0.041 (5)	0.058 (5)	0.001 (3)	0.013 (4)	0.005 (4)
C23	0.039 (4)	0.031 (4)	0.032 (4)	0.001 (3)	0.010 (3)	0.004 (3)
C24	0.026 (4)	0.028 (4)	0.035 (4)	−0.001 (3)	0.000 (3)	0.004 (3)
O1	0.082 (8)	0.075 (9)	0.095 (9)	−0.024 (7)	−0.026 (7)	0.007 (7)

### (2) Geometric parameters (Å, º)

**Table:**

Cu1—N5	1.969 (6)	Cu2—N10	1.961 (7)
Cu1—N3	2.008 (6)	Cu2—N7	2.010 (6)
Cu1—N2	2.025 (6)	Cu2—N6	2.027 (6)
Cu1—N1	2.049 (6)	Cu2—N8	2.046 (6)
Cu1—N4	2.140 (6)	Cu2—N9	2.123 (7)
S1—C12	1.626 (7)	S3—C24	1.633 (7)
S2—C11	1.626 (7)	S4—C23	1.615 (8)
N1—C10	1.474 (9)	N6—C17	1.330 (9)
N1—C9	1.489 (9)	N6—C13	1.337 (9)
N1—C8	1.495 (10)	N7—C18	1.470 (9)
N2—C6	1.464 (9)	N7—C19	1.496 (9)
N2—C7	1.477 (9)	N8—C20	1.474 (9)
N3—C1	1.324 (9)	N8—C22	1.478 (9)
N3—C5	1.362 (9)	N8—C21	1.489 (9)
N4—C11	1.143 (9)	N9—C24	1.147 (9)
N5—C12	1.146 (9)	N10—C23	1.161 (9)
C1—C2	1.361 (10)	C13—C14	1.398 (11)
C2—C3	1.379 (11)	C14—C15	1.378 (13)
C3—C4	1.378 (10)	C15—C16	1.365 (11)
C4—C5	1.397 (9)	C16—C17	1.381 (10)
C5—C6	1.474 (10)	C17—C18	1.509 (10)
C7—C8	1.497 (11)	C19—C20	1.491 (10)
			
N5—Cu1—N3	95.4 (2)	N10—Cu2—N7	161.1 (3)
N5—Cu1—N2	161.6 (3)	N10—Cu2—N6	94.9 (2)
N3—Cu1—N2	81.1 (2)	N7—Cu2—N6	80.7 (2)
N5—Cu1—N1	93.7 (2)	N10—Cu2—N8	93.4 (3)
N3—Cu1—N1	162.1 (2)	N7—Cu2—N8	85.2 (2)
N2—Cu1—N1	85.3 (2)	N6—Cu2—N8	158.7 (2)
N5—Cu1—N4	97.4 (2)	N10—Cu2—N9	97.6 (3)
N3—Cu1—N4	93.7 (2)	N7—Cu2—N9	101.2 (3)
N2—Cu1—N4	100.9 (2)	N6—Cu2—N9	100.1 (2)
N1—Cu1—N4	100.4 (2)	N8—Cu2—N9	98.2 (3)
C10—N1—C9	108.2 (6)	C17—N6—C13	119.3 (6)
C10—N1—C8	110.5 (6)	C17—N6—Cu2	115.3 (4)
C9—N1—C8	109.8 (6)	C13—N6—Cu2	125.4 (5)
C10—N1—Cu1	112.9 (5)	C18—N7—C19	114.0 (6)
C9—N1—Cu1	110.0 (5)	C18—N7—Cu2	110.5 (4)
C8—N1—Cu1	105.4 (4)	C19—N7—Cu2	108.5 (4)
C6—N2—C7	116.9 (6)	C20—N8—C22	110.5 (6)
C6—N2—Cu1	109.0 (4)	C20—N8—C21	110.1 (6)
C7—N2—Cu1	109.0 (5)	C22—N8—C21	107.8 (6)
C1—N3—C5	119.5 (6)	C20—N8—Cu2	105.8 (4)
C1—N3—Cu1	126.2 (5)	C22—N8—Cu2	110.0 (5)
C5—N3—Cu1	114.0 (4)	C21—N8—Cu2	112.7 (5)
C11—N4—Cu1	163.3 (6)	C24—N9—Cu2	172.2 (7)
C12—N5—Cu1	165.5 (6)	C23—N10—Cu2	169.6 (6)
N3—C1—C2	123.0 (7)	N6—C13—C14	121.2 (8)
C1—C2—C3	118.6 (7)	C15—C14—C13	118.8 (7)
C4—C3—C2	119.9 (6)	C16—C15—C14	119.4 (7)
C3—C4—C5	118.8 (7)	C15—C16—C17	119.0 (8)
N3—C5—C4	120.2 (6)	N6—C17—C16	122.3 (7)
N3—C5—C6	115.4 (6)	N6—C17—C18	115.2 (6)
C4—C5—C6	124.4 (6)	C16—C17—C18	122.5 (7)
N2—C6—C5	109.4 (6)	N7—C18—C17	109.5 (6)
N2—C7—C8	107.3 (6)	C20—C19—N7	106.7 (6)
N1—C8—C7	110.4 (6)	N8—C20—C19	109.7 (6)
N4—C11—S2	177.8 (7)	N10—C23—S4	178.3 (8)
N5—C12—S1	179.1 (7)	N9—C24—S3	178.0 (7)
			
C5—N3—C1—C2	1.0 (10)	C17—N6—C13—C14	0.5 (10)
Cu1—N3—C1—C2	−172.5 (5)	Cu2—N6—C13—C14	−178.3 (5)
N3—C1—C2—C3	−0.3 (10)	N6—C13—C14—C15	0.9 (11)
C1—C2—C3—C4	−0.5 (10)	C13—C14—C15—C16	−1.5 (12)
C2—C3—C4—C5	0.6 (10)	C14—C15—C16—C17	0.8 (11)
C1—N3—C5—C4	−0.9 (9)	C13—N6—C17—C16	−1.3 (10)
Cu1—N3—C5—C4	173.4 (5)	Cu2—N6—C17—C16	177.6 (5)
C1—N3—C5—C6	−179.3 (6)	C13—N6—C17—C18	−179.2 (6)
Cu1—N3—C5—C6	−5.1 (7)	Cu2—N6—C17—C18	−0.3 (7)
C3—C4—C5—N3	0.1 (10)	C15—C16—C17—N6	0.7 (11)
C3—C4—C5—C6	178.4 (6)	C15—C16—C17—C18	178.4 (7)
C7—N2—C6—C5	−160.1 (6)	C19—N7—C18—C17	154.3 (6)
Cu1—N2—C6—C5	−35.9 (7)	Cu2—N7—C18—C17	31.8 (7)
N3—C5—C6—N2	27.4 (8)	N6—C17—C18—N7	−20.8 (8)
C4—C5—C6—N2	−150.9 (7)	C16—C17—C18—N7	161.3 (6)
C6—N2—C7—C8	162.0 (6)	C18—N7—C19—C20	−162.4 (6)
Cu1—N2—C7—C8	38.0 (7)	Cu2—N7—C19—C20	−38.8 (7)
C10—N1—C8—C7	163.1 (6)	C22—N8—C20—C19	76.4 (8)
C9—N1—C8—C7	−77.6 (7)	C21—N8—C20—C19	−164.6 (6)
Cu1—N1—C8—C7	40.9 (7)	Cu2—N8—C20—C19	−42.6 (7)
N2—C7—C8—N1	−53.3 (8)	N7—C19—C20—N8	54.8 (8)

### (2) Hydrogen-bond geometry (Å, º)

**Table:**

*D*—H···*A*	*D*—H	H···*A*	*D*···*A*	*D*—H···*A*
C6—H6*A*···S2^i^	0.99	2.94	3.786 (8)	144
C8—H8*A*···S3	0.99	2.95	3.785 (8)	143
C8—H8*B*···O1^ii^	0.99	2.65	3.565 (15)	154
C9—H9*A*···S1^i^	0.98	2.92	3.881 (9)	166
C9—H9*B*···N5	0.98	2.68	3.186 (10)	112
N2—H2*N*···S3	0.91 (7)	2.68 (7)	3.546 (7)	161 (9)
C14—H14···S2^iii^	0.95	2.87	3.810 (9)	170
C18—H18*A*···S4^i^	0.99	3.00	3.932 (8)	158
C19—H19*B*···S3^i^	0.99	3.01	3.764 (8)	133
C20—H20*B*···O1^iii^	0.99	2.59	3.475 (16)	149
C21—H21*C*···N9	0.98	2.64	3.186 (10)	116
C22—H22*A*···N10	0.98	2.65	3.157 (11)	112
C22—H22*B*···S4^i^	0.98	2.94	3.912 (8)	170
N7—H7*N*···S2^i^	0.91 (6)	2.83 (7)	3.623 (7)	146 (6)

Symmetry codes: (i) *x*, *y*+1, *z*; (ii) *x*, −*y*+1, *z*+1/2; (iii) −*x*+1/2, *y*+1/2, −*z*+1/2.
